# On the interaction of toxic Heavy Metals (Cd, Hg, Pb) with graphene quantum dots and infinite graphene

**DOI:** 10.1038/s41598-017-04339-8

**Published:** 2017-06-21

**Authors:** Ivan Shtepliuk, Nuala M. Caffrey, Tihomir Iakimov, Volodymyr Khranovskyy, Igor A. Abrikosov, Rositsa Yakimova

**Affiliations:** 1 Department of Physics, Chemistry and Biology, Linköping University, SE-58183 Linköping, Sweden; 2 Frantsevich Institute for Problems of Materials Science, NASU, 03680 Kyiv-142, Ukraine; 3 School of Physics and CRANN, Trinity College, Dublin 2, Ireland; 4 Materials Modeling and Development Laboratory, National University of Science and Technology “MISIS”, Moscow, Russia

## Abstract

The promise of graphene and its derivatives as next generation sensors for real-time detection of toxic heavy metals (HM) requires a clear understanding of behavior of these metals on the graphene surface and response of the graphene to adsorption events. Our calculations herein were focused on the investigation of the interaction between three HMs, namely Cd, Hg and Pb, with graphene quantum dots (GQDs). We determine binding energies and heights of both neutral and charged HM ions on these GQDs. The results show that the adsorption energy of donor-like physisorbed neutral Pb atoms is larger than that of either Cd or Hg. In contrast to the donor-like behavior of elemental HMs, the chemisorbed charged HM species act as typical acceptors. The energy barriers to migration of the neutral adatoms on GQDs are also estimated. In addition, we show how the substitution of a carbon atom by a HM adatom changes the geometric structure of GQDs and hence their electronic and vibrational properties. UV-visible absorption spectra of HM-adsorbed GQDs vary with the size and shape of the GQD. Based on our results, we suggest a route towards the development of a graphene-based sensing platform for the optical detection of toxic HMs.

## Introduction

As a two-dimensional material with unique electronic properties, graphene is anticipated to be a key element in novel sensing platforms^1^. Three main attributes of graphene make it attractive for such applications: (i) a surface area of 2600 m^2^·g^−1^, thereby providing a large platform for surface chemistry^2^, (ii) its ability to act as either a reducing agent (electron donor) or an oxidizing agent (electron acceptor)^3^ and (iii) low Johnson noise, even at low charge densities and at room temperature, as a result of its high conductivity and lack of a band gap^4^. Combined, these properties mean that graphene-based sensors are capable of detecting changes in the local charge concentration of less than single electron^4^, and thus the presence of small concentrations of surface functional groups and adsorbates.

Further functionality can be achieved by considering zero-dimensional graphene quantum dots (GQDs). Due to the quantum confinement effect, GQDs exhibit a size-dependent band gap^5^, which results in a variable photoluminescence emission^6^. The adsorption of certain ions or molecules can modify the size of the band gap and thereby cause a photoluminescence quenching or enhancement effect. Such a change in the optical properties can be effectively detected and so harnessed to create sensors.

Driven by environmental and health concerns, there is currently a strong impetus towards the development of fast, inexpensive commercial sensors for the detection of hazardous heavy metals^7^, such as Cd, Hg and Pb. Beyond a certain limit, exposure to these elements can cause severe detrimental effects to human health and to the environment in general^8^. Cd is classified as a human carcinogen, resulting in lung cancer as well as various renal problems. Hg exposure is a major public health concern, causing widespread problems to the nervous, digestive and immune systems as well as to the development a fetus. Although most exposure to Hg occurs by eating contaminated fish and shellfish, elemental mercury vapors can also be inhaled by workers in industrial processes. Lead poisoning affects the central nervous system, and is particularly harmful to children. Its widespread use in paint and water pipes mean that is significant public health concern in many parts of the world. According to the World Health Organization reports^9^, the maximal permissible limits of Cd, Hg and Pb in drinking water are estimated to be 3, 1 and 10 μg/L, respectively.

The relative toxicity of these ions also depends strongly on their oxidation state^10^. This is due to their ability to form salts with different solubilities^11–13^. For example, Hg^1+^ (mercurous) and Hg^2+^ (mercuric) valence states mercury form different inorganic salts. Hg^2+^ - containing salts are more soluble and hence more dangerous than Hg^1+^-containing salts. Thus, the ability to detect very low concentrations of these non-biocompatible heavy metals in different oxidation states is vital.

To date, a number of graphene-based sensors for detection of heavy metals have been proposed^7, 14–27^. The fundamental operational principle of these sensors is the chemical interaction between the adsorbate (heavy metal ions) and the graphene electrode, which strongly modifies the measurable electrical properties of the graphene-adsorbate system, such as the electric current or potential. The resulting electrochemical signal can then be used to quantitatively determine the concentration of toxic heavy metals on the surface. Several experimental investigations have considered how charged ions of Cd, Hg and Pb interact with carbon-based materials, such as functionalized graphene, graphene oxide and carbon nanotubes^28–41^.

GQDs are, to date, not as well investigated for sensing applications as those based on functionalized graphene. Here, we explore the adsorption of neutral, monovalent and divalent ions of Cd, Hg and Pb on both graphene quantum dots and infinite graphene, in order to determine their suitability as sensors for heavy metal detection.

## Results and Discussion

The current section is organized as follows. First, we describe the geometric and energetic features associated with the adsorption of heavy metal atoms and ions on C_54_H_18_ graphene quantum dots. We then discuss the electronic properties of the interacting systems. Next, we consider the nature of the mobility and clustering of heavy metal adatoms on graphene quantum dots and the effects of substitutional impurities on the structural and physical properties of GQDs. Finally, we show that the interaction between the HM atoms and the GQD depends on the size and edge of the latter, and determine how the latter will influence the UV-visible adsorption spectra. Our results confirm a possibility to develop realistic optical sensing applications for HMs detection.

**Figure 1:**
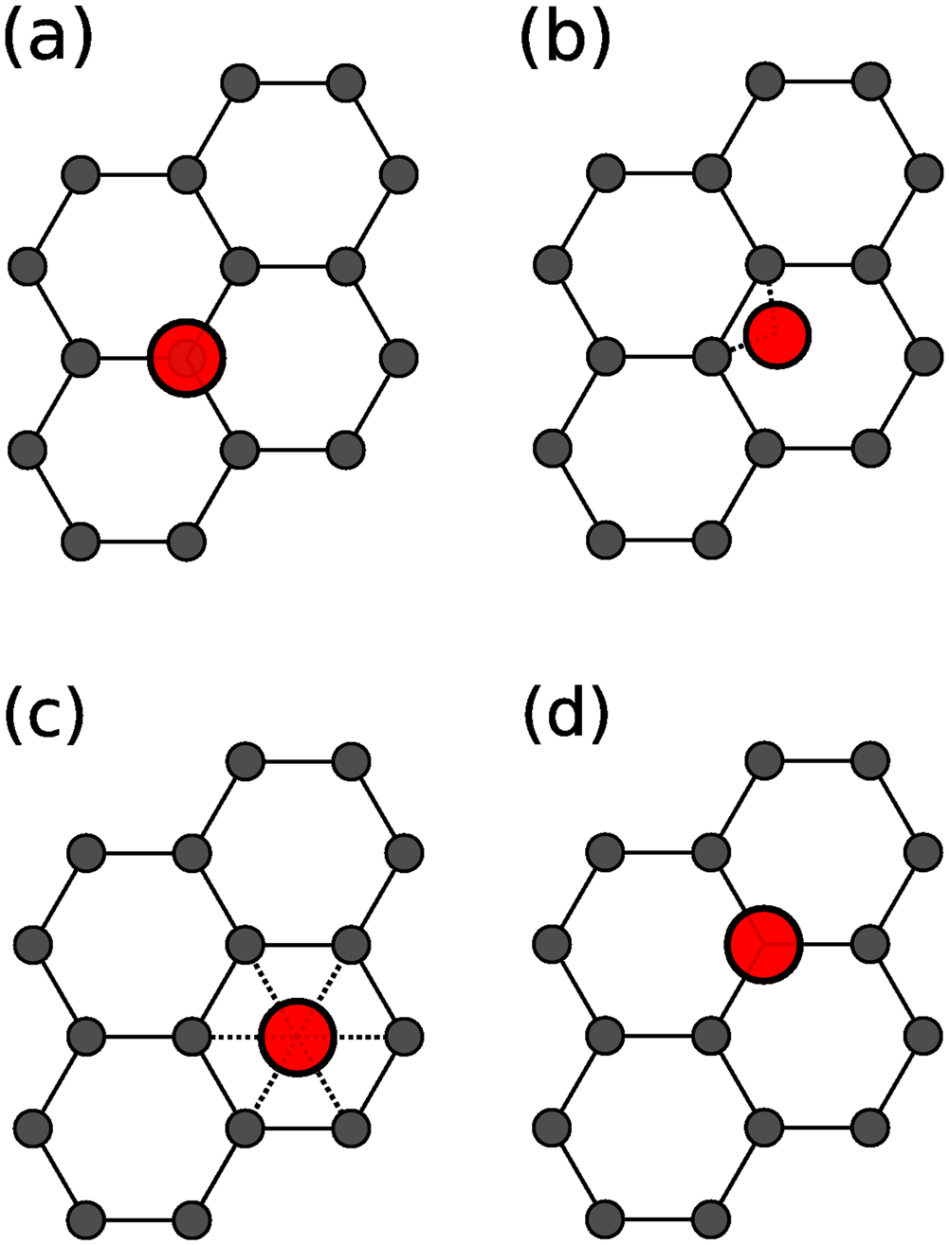
Illustration of the high symmetry adsorption sites. Neutral atoms and charged ions of heavy metals are placed at the (**a**) on-top (*T*), (**b**) bridge (*B*), and (**c**) hollow (*H*) sites on the hexagonal cell, as well as, on a defect (*D*) site (**d**) of graphene sheet (substitutional defect).

As the adsorption site of the adatom is known to influence the electronic properties^23^, here we explore four adsorption sites, namely the hollow site (*H*), where the adatom sits in the center of the hexagonal ring, the bridge site (*B*), where atoms are placed at the center of a C-C bond, the top site (*T*), where the adatom is adsorbed directly on top of a carbon atom and a defect site (*D*), where the adatom is adsorbed on top of a carbon vacancy in the GQD. These configurations are illustrated in Fig. 1.

### Binding site and binding energies

#### Neutral atoms

**Figure 2:**
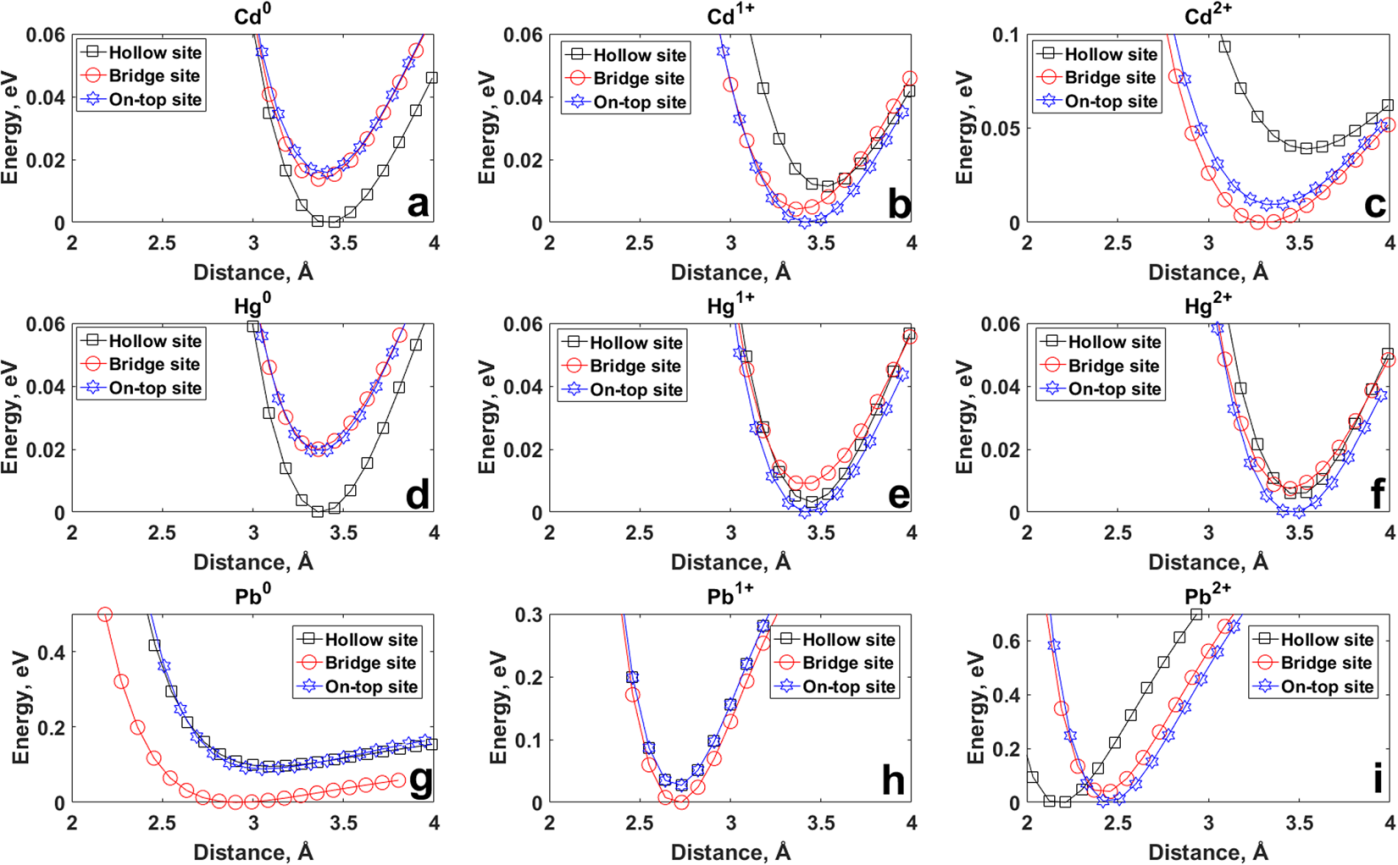
Potential energy curves for neutral atoms: (**a**) Cd°, (**d**) Hg° and (**g**) Pb°; for monovalent ions: (**b**) Cd^1+^, (**e**) Hg^1+^ and (**h**) Pb;^1+^ and for divalent ions: (**c**) Cd^2+^, (**f**) Hg^2+^ and (**i**) Pb^2+^on C_54_H_18_ quantum dots (top-site position, hollow site position, bridge site position). The energy is given relative to the largest negative value of the total energy of the system over the entire scanning range.

We determine the binding site and binding height of the adatoms by plotting the total energy as a function of their distance from a zigzag-edged C_54_H_18_ GQD. Such as large GQD was chosen to minimize the effect on the edge on the adsorption location, and thereby to approximate infinite graphene. The results are shown in Fig. 2(a,d,g) for Cd°, Hg° and Pb°, respectively.

We find that the binding height and binding position depend on the adatoms. The most energetically-favorable binding site for the adsorption of Cd° and Hg° on the GQD is the hollow site, while that of the Pb° is the bridge site. These results are summarized in Table 1. The equilibrium adatom heights are found to be 3.441 Å for Cd°, 3.322 Å for Hg° and 2.904 Å for Pb°, i.e., the binding height follows the sequence Cd° > Hg° > Pb°. This trend is in agreement with other calculations in the literature^42^.

**Table 1:** Binding energy, equilibrium distance and charge transfer of neutral and charged Cd, Hg and Pb species on C_54_H_18_.

Metal	Preferred adsorption site	Binding energy, eV	Equilibrium distance, Å	Mulliken charge transfer	Nature of interaction
Cd°	*Hollow*	0.170	3.441	0.09	Physisorption
Cd^1+^	*Top*	3.145	3.361	−0.99	Chemisorption
Cd^2+^	*Bridge*	11.663	3.276	−1.87	Chemisorption
Hg°	*Hollow*	0.161	3.322	0.09	Physisorption
Hg^1+^	*Top*	4.426	3.428	−0.99	Chemisorption
Hg^2+^	*Top*	14.563	3.390	−1.94	Chemisorption
Pb°	*Bridge*	0.199	2.904	0.24	Physisorption
Pb^1+^	*Bridge*	2.024	2.770	−0.16	Chemisorption
Pb^2+^	*Hollow*	7.0549	2.216	−0.93	Chemisorption

The charge transfer is calculated using the Mulliken charge analysis. A negative value implies that charge is transferred from the graphene quantum dots to the adatom.

The smallest binding energies are found for Cd° and Hg° adatoms, at 0.170 eV and 0.161 eV, respectively. The binding energy for Pb° adatoms is found to be larger, at 0.199 eV. Adatoms are generally considered to be physisorbed on a surface when their binding energy is lower than 0.5 eV per adsorbed species^43^. The calculated adsorption energies of neutral Cd°, Hg° and Pb° indicate the physisorption nature of the interaction between neutral HMs and hydrogen-terminated GQDs.

The charge transfer to or from the adatom was calculated using Mulliken charge analysis^44^. A small charge transfer of approximately 0.09e occurs from the Cd° and Hg° adatoms to C_54_H_18_ quantum dot. In contrast, the Pb° atom donates approximately 0.24e to the carbon atoms. The difference in the charge transfer between GQD and the Cd°, Hg° and Pb° adatoms can be understood in terms of the ionization energy of the metal^45^. The ionization potential of Pb° is approximately equal to 7.41 eV^46, 47^, whereas Cd and Hg have larger ionization energies of 8.99 eV and 10.43 eV^46, 47^, respectively. Thus, the Pb° transfers electrons more easily to the GQD than Cd° and Hg° adatoms.

### Charged ions

Figure 2(b,e,h and c,f,i) show the total energy for monovalent and divalent ionic species of the heavy metals (Cd^1+^, Cd;^2+^ Hg^1+^, Hg;^2+^ and Pb^1+^, Pb^2+^) adsorbed on the C_54_H_18_ as a function of the binding height. Unlike the weakly-bound neutral adatoms, the charged ions are now chemisorbed on the GQD. In all cases, the binding position changes for the charged ions compared to their neutral counterparts. Cd^1+^ and Cd^2+^ are now preferentially adsorbed on the *T* and *B* site, respectively. The Hg ions adsorb on the *T*-site, compared to the *H*-site for the neutral adatom. While the monovalent Pb adatom remains on the *B*-site, as for the neutral adatom, it switches to the *H*-site for the divalent adatom. As expected, the positively charged ions reverse the direction of charge transfer compared to the charge-donating neutral adatoms. This amounts to approximately 0.9e^−^ for Cd^1+^, 1.8e^−^ for Cd^2+^, 0.9e^−^ for Hg^1+^, 2e^−^ for Hg^2+^, 0.2e^−^ for Pb^1+^ and 0.9e^−^ for Pb^2+^, as shown in Table 1.

The equilibrium distances of the monovalent and divalent ions from the graphene surface descend in the same sequence: Hg^1+^ > Cd^1+^ > Pb^1+^ and Hg^2+^ > Cd^2+^ > Pb^2+^ (see Table 1). This is in contrast to the sequence for the neutral atoms (Cd° > Hg° > Pb°). Similarly, the sequence of binding energies changes for the ions compared to the neutral atoms: from Pb° > Cd° > Hg° to Hg^1+/2+^ > Cd^1+/2+^ > Pb^1+/2+^. This may be ascribed to the electronegativity trends of the metal ions^48^.

#### Supercell approach

As a comparison, we also determined the binding position and height of these adatoms on extended graphene, modelled using the supercell approach. The main trends are in broad agreement with those determined for GQDs, i.e., the binding site of the Cd and Hg adatoms is also found to be the hollow-site, while Pb binds preferentially on the top site, with a small energy difference between it and the bridge site (8 meV) and a significantly larger energy difference between it and the hollow site (119 meV). The binding height of the Cd adatom is found to be 3.46 Å. This is slightly larger than that found using the GQD approach (3.441 Å). A similar slight increase in binding height occurs for the case of a Hg (Pb) adatom, from 3.322 Å (2.904 Å) to 3.33 Å (2.96 Å). These differences can be due to the influence of the finite size of the GQDs, their hydrogened edges^49^, as well as the different DFT approximations involved in both calculations.

The supercell calculations give increased binding energies for Cd and Hg compared to the calculations for the GQD: 0.218 eV and 0.246 eV for Cd and Hg, respectively, compared to 0.170 eV and 0.161 eV. The binding energy of Pb is also increased to 0.372 eV. Due to the unpaired electrons of the Pb adatom, it exhibits a magnetic moment of 1.78 µ_B_ when adsorbed on the graphene surface. The non-magnetic solution is 0.385 eV higher in energy.

**Figure 3:**
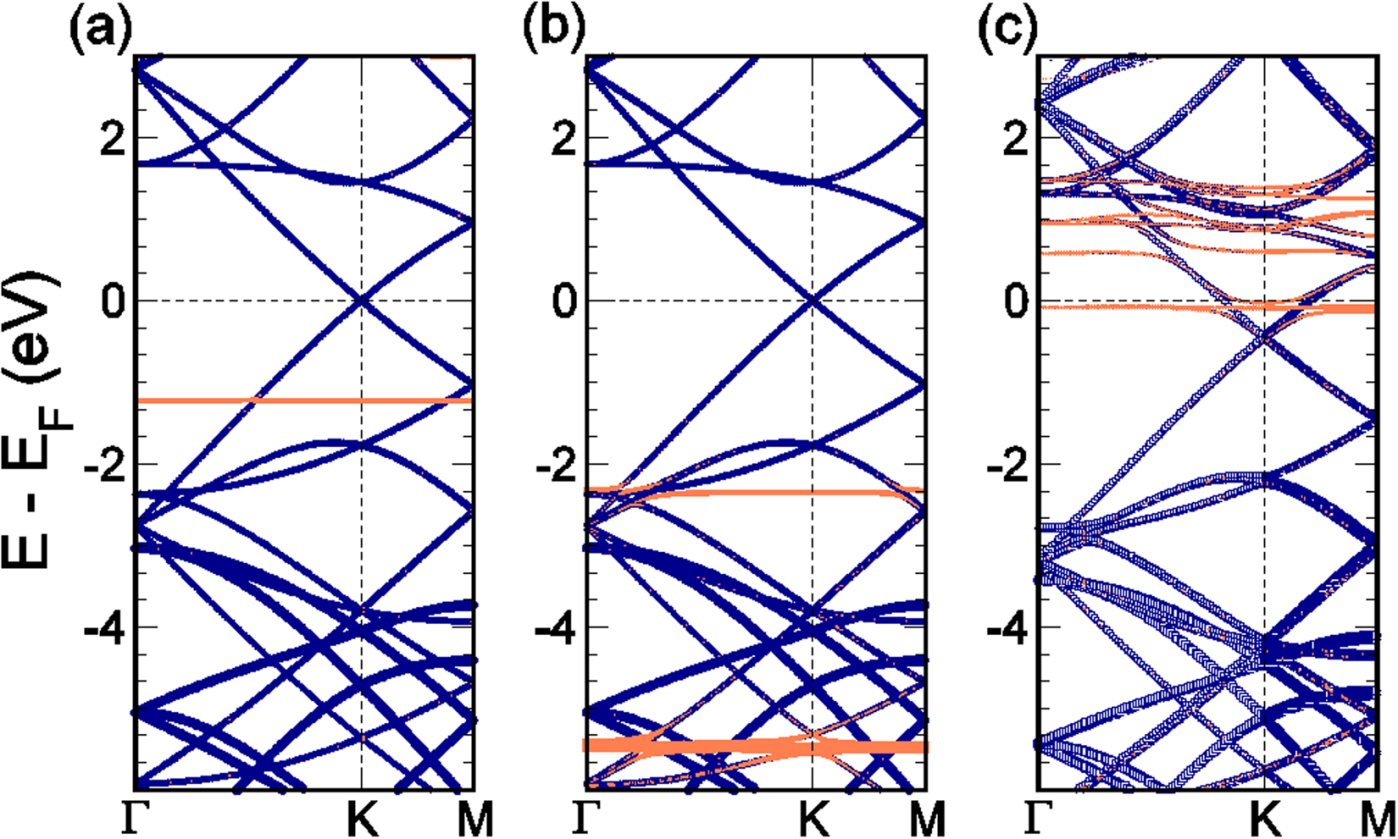
Band structure of (**a**) Cd, (**b**) Hg and (**c**) Pb atoms adsorbed on a 4 × 4 graphene supercell. The purple lines denote those bands with a large graphene character, while the orange lines have a higher character from the metal adatom. This band character was determined by projecting onto the atomic orbitals of the respective atoms. The band thickness represents the magnitude of the overlap.

In agreement with the GQD calculations, the supercell calculations also find minimal charge transfer between the physisorbed adatoms and the graphene sheet. We find Cd (Hg) transfers 0.02e (0.05e) to the carbon atoms, while the Pb atom transfers 0.27e to the graphene sheet. The effects of this charge transfer can be seen in the shift of the graphene Dirac cone towards higher binding energies after Pb adsorption. This is shown in Fig. 3. While for Cd and Hg, the Dirac point remains at the Fermi level (Fig. 3a and b)), when Pb is adsorbed on graphene the Dirac point is shifted to 0.48 eV below the Fermi level, due to the strong *n*-doping from the adsorbant.

#### Frontier molecular orbitals

**Figure 4:**
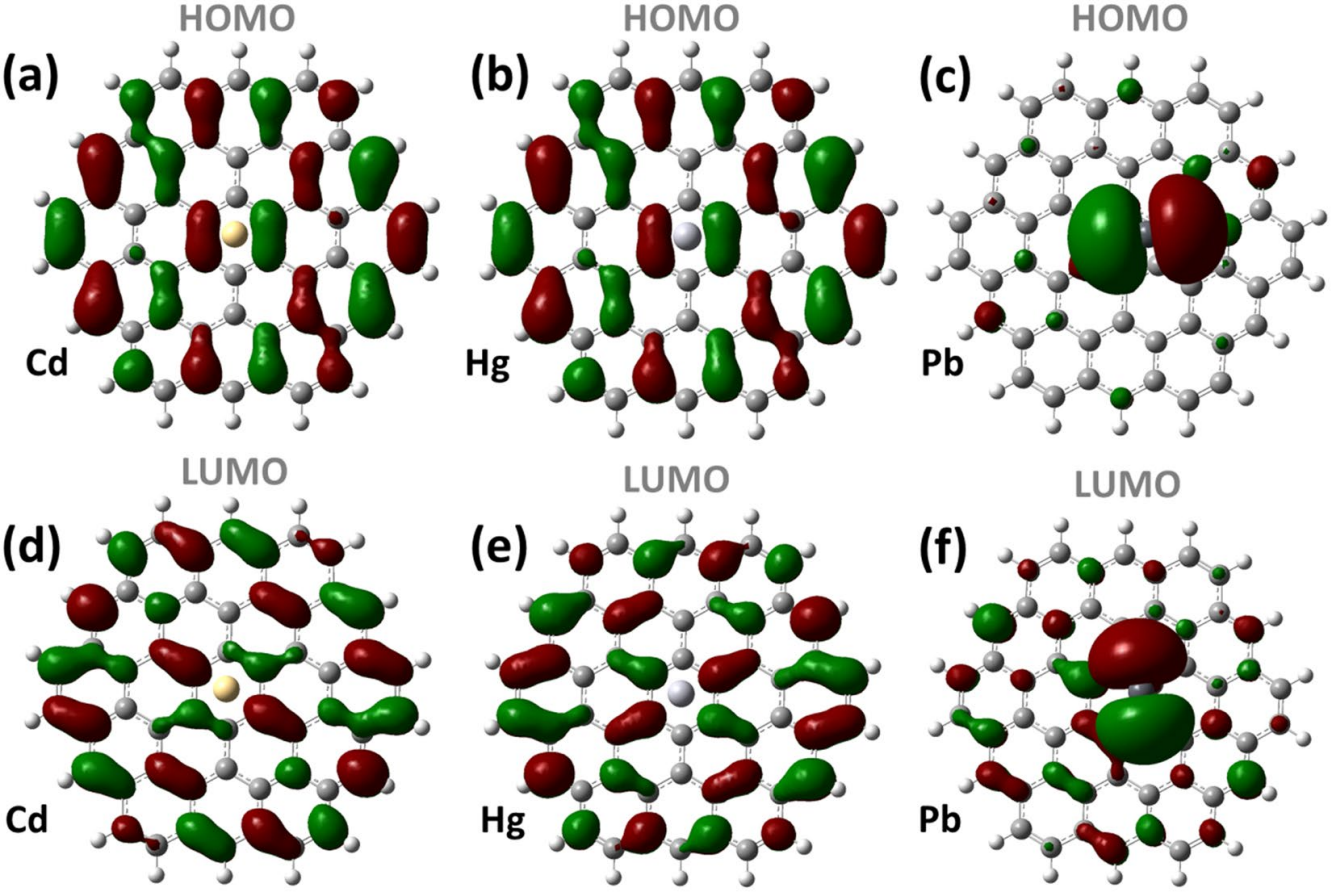
Molecular orbitals for C_54_H_18_ GQDs interacted with heavy metals: HOMO (**a**) and LUMO (**d**) of Cd@C_54_H_18_; HOMO (**b**) and LUMO (**e**) of Hg@C_54_H_18_; and HOMO (**c**) and LUMO (f) of Pb@C_54_H_18_.

In order to understand the electronic interaction between the neutral heavy metal adsorbants and graphene quantum dots, we plot in Fig. 4 the frontier molecular orbitals for the C_54_H_18_ GQDs after the adsorption of neutral heavy metal adatoms. It is clear that the Cd° and Hg° atoms do not contribute to the formation of both LUMO and HOMO (see also in Fig. 4a and b, respectively). Instead, they are completely  delocalized over the surface of the GQDs, confirming the physisorption character of the adsorption processes. This is in agreement with the small amount of charge transfer that occurs for these two atoms [c.f. Table 1]. Furthermore, the HOMO-LUMO gap of the Cd@C_54_H_18_ and Hg@C_54_H_18_ quantum dots is similar to that of the isolated C_54_H_18_ (~2.88 eV), which also suggests a very weak interaction between the two. In contrast, Pb@C_54_H_18_ exhibits qualitatively different behavior (Fig. 4c). In particular, the HOMO and LUMO are shared by both the adsorbate and the quantum dots, indicating a strong hybridization between the two (Fig. 4c and f). As a result the HOMO-LUMO gap for Pb@C_54_H_18_ is reduced to 0.74 eV.

### Mobility of the heavy metal adatom on GQD

**Figure 5:**
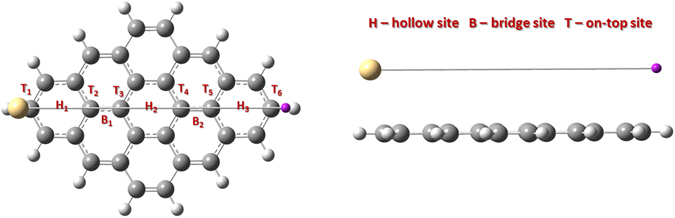
Path of the surface motion of the neutral generic HM atom on C_30_H_16_ GQD.

**Figure 6:**
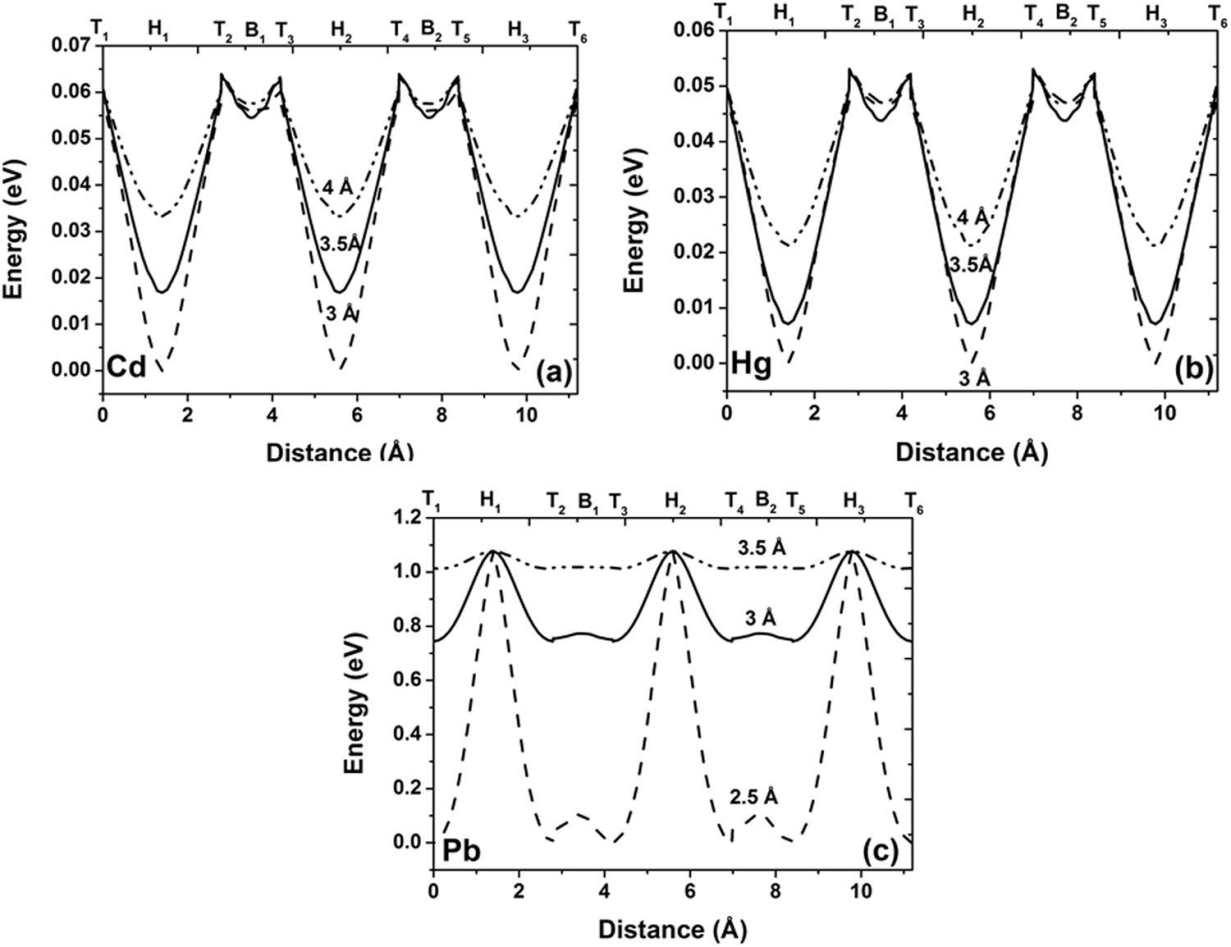
Potential energy curves describing the in-plane motion of the neutral heavy metal atoms along the C_30_H_16_ GQDs for: (**a**) cadmium, (**b**) mercury and (**c**) lead. *T*_1_–*T*_6_ denote the potential energy for heavy metal adatom on on-top site position, *B*_1_ and *B*_2_ represent the bridge site positions and hollow site positions are marked as *H*_1_–*H*_3_. The potential energy of the interacting system at the each point of the diffusion pathway is set by the lowest value of the total energy of the system over the entire scanning range.

Here we investigate the tendency of the heavy metal adatoms to diffuse across the surface of a C_30_H_16_ GQD, by determining the total energy of the interacting system as the atom is moved over the surface. To understand the mobility specificity and to avoid time consuming calculations we consider a motion of HMs on smaller GQD than C_54_H_18_ (described in previous sections), which still retains the attributes of the bigger one. As in the case of the C_54_H_18_, the chosen path for total energy calculations on C_30_H_16_ GQD passes over all three high-symmetry positions, namely the *T*, *H* and *B* sites (Fig. 5). The adatom is moved in steps of 0.05 Å along the diffusion pathway at several values of the fixed height (3 Å, 3.5 Å and 4 Å). The length of the path is equal to 11.17 Å. As can be seen from Fig. 6a and b, the potential energy curves of cadmium and mercury on the GQDs are similar and have several energetic minima corresponding to the stable hollow sites. The migration of cadmium (or mercury) from a hollow site to a bridge site requires overcoming an energy barrier as high as 45 meV, when the adatom is located 3.5 Å above the graphene quantum dot. On the other hand, an energy barrier of only 5 meV exists between the *B*_1_ site and the *T*_2_-site. As expected, a decrease in the binding height from 4 Å to 3 Å leads to an increase in the activation energies for surface mobility.

In contrast, the potential energy curves associated with the mobility of a Pb atom at different heights (Fig. 6c) are characterized by high energy maxima located at hollow site positions and deep minima at top and bridges sites. Pb, at a height of 3 Å above the surface, will therefore prefer to diffuse along the C-C bond, with a low energy barrier of just 20 meV. In order to migrate across the *H*-site a Pb atom must overcome a high energy barrier exceeding 0.3 eV, when the Pb adatom is located 3 Å above the graphene quantum dot. Our findings are in good agreement with experimental results, which found that Pb adatoms on graphene have extremely low barriers to surface motion −35 meV at 30 K and 70 meV at 70 K^50^.

**Figure 7:**
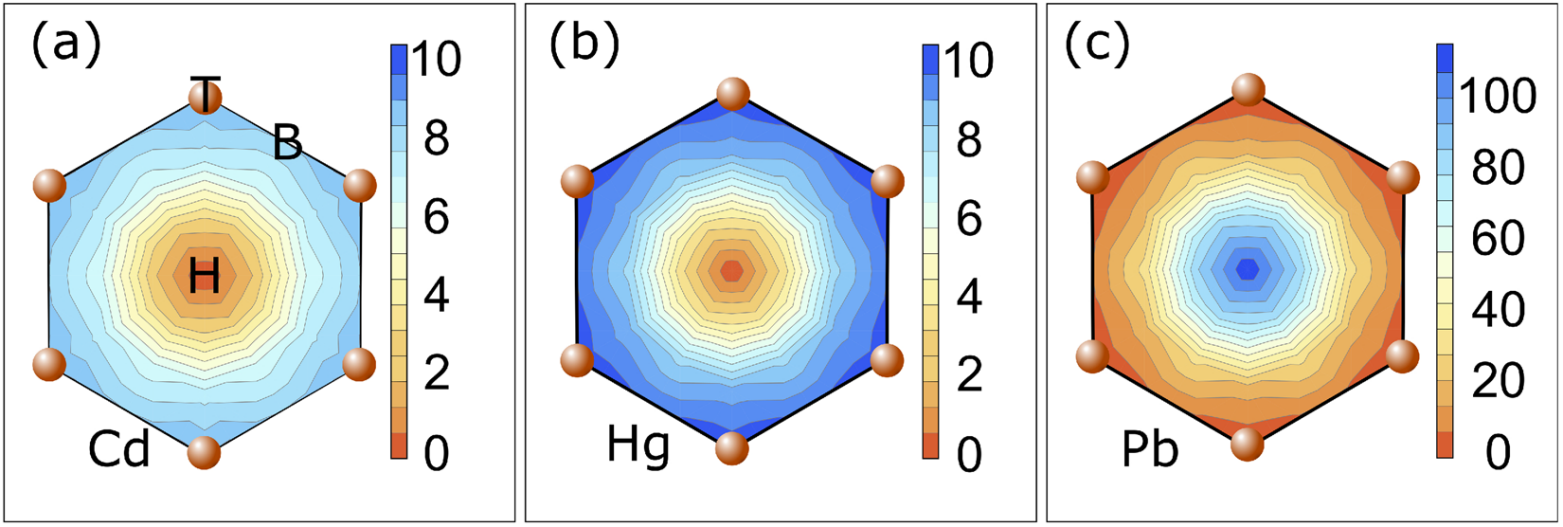
Total energy (in meV) as a function the (**a**) Cd, (**b**) Hg, and (**c**) Pb adatom position on a graphene hexagon, calculated for a 4 × 4 supercell. In all cases, the minimum total energy is set to 0 eV. Note the change in scale for (**c**).

The energy barriers between the high-symmetry positions on extended graphene, calculated by VASP, are similar to those calculated for the GQDs. The total energy as a function of the adatom binding position on an extended graphene sheet is shown in Fig. 7 for Cd, Hg, and Pb, respectively. The total energy was calculated at 9 unique adatom binding positions within the graphene hexagon, including the three high symmetry points, *T*, *B* and *H*, as well as at two points along the lines connecting the high symmetry points. The height of the adatom above the graphene surface was allowed to relax at each in-plane position. In comparison to the results obtained on the GQDs, the supercell calculations result in smaller energy barriers between adsorption sites, due to the out-of-plane relaxation allowed at each step. Otherwise, the two methods are in good agreement.

### Metal clustering on graphene QDs

In the previous sections, we showed how heavy metal adatoms adsorb and diffuse on C_30_H_16_ and C_54_H_18_ GQDs as well as on an infinite graphene sheet. We found that both Cd and Hg are weakly bonded to the graphene quantum dot and furthermore, that the energy differences between the three high-symmetry binding sites are quite small. This would suggest that these atoms could easily diffuse across the surface and eventually form metal islands or clusters. If this occurs, it could lead to significant changes in the properties of graphene, as has been previously reported for Pt and transition metal adatoms^51, 52^. Therefore, in order to gain a complete picture of the behavior of heavy metals on the graphene surface, we study the nature of the interaction between *n* (*n* = 2… 4) adatoms and a C_16_H_10_ GQD. The choice of the small and simplest C_16_H_10_ QQD is justified because we are mainly interested in the search of the critical distance when the metal-metal attraction becomes dominating. At this nano-scale, this distance cannot exceed the HM isomer bond lengths in the gas-phase and thus even the simplest GQD can be representative of GQD family to understand the specificity of the metal clustering on the *sp* ^2^ bonded conjugated hexagonal rings. The effect of the GQD size on the metal-metal interaction is expected to be minor.

**Figure 8:**
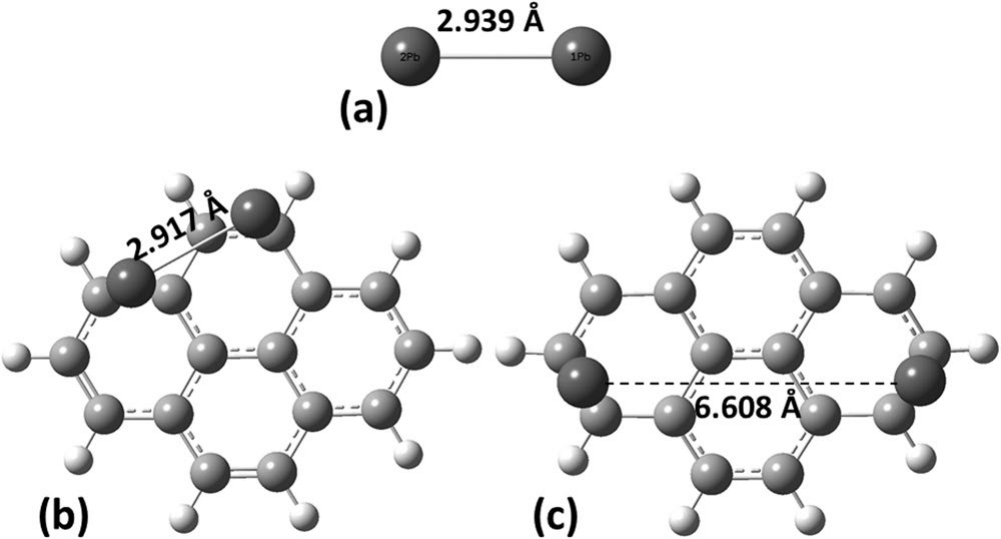
Equilibrium configuration of Pb_2_ dimer in vacuum (**a**) and optimized geometries for two Pb atoms on C_16_H_10_ GQDs pre-adsorbed at short (**b**) and long (**c**) initial distances from one another, respectively.

First, we explore the formation of heavy metal dimers, i.e. a cluster consisting of two adatoms. There are two competing mechanisms here: (1) the formation of bonds between the metal atoms and graphene and (2) the formation of bonds between the two metal atoms. The relative strength of these interactions will be dependent on the distance between the adatoms on the surface of graphene. The experimental bond lengths for isolated Cd_2_ and Hg_2_ dimers are in the range of 3.78–4.33 Å^53–55^ and 3.605–3.69 Å^56–58^, respectively. The bond length of a stable Pb_2_ dimer is 2.93 Å^59^. Our calculations find the equilibrium distances for isolated Cd_2_, Hg_2_ and Pb_2_ dimers to be 4.131 Å, 3.968 Å and 2.939 Å, respectively. Apart from the equilibrium distance for Hg, these values are in good agreement with the experimental parameters. To determine the tendency of two heavy metal adatoms adsorbed on graphene to cluster, we consider two initial geometrical configurations where the adatoms have been separated by different distances. After optimization, we find that two adatoms, initially placed far from each other, tend to bind to graphene quantum dot, rather than each other. On the other hand, two adatoms placed close to each other form dimer structures, with equilibrium distances of 3.691 Å, 3.759 Å and 2.917 Å for Cd_2_, Hg_2_ and Pb_2_, respectively. For the case of Cd and Hg, the total energy of the system is lower when the two adatoms are far from each other, by 24 meV per atom. This suggests that despite a low diffusion barrier, these adatoms should not tend to form islands on graphene. On the other hand, the formation of Pb dimers on the graphene surface is highly favourable, with an energy gain of 1.046 eV per Pb atom. The relaxed structures are shown in Fig. 8.

**Figure 9:**
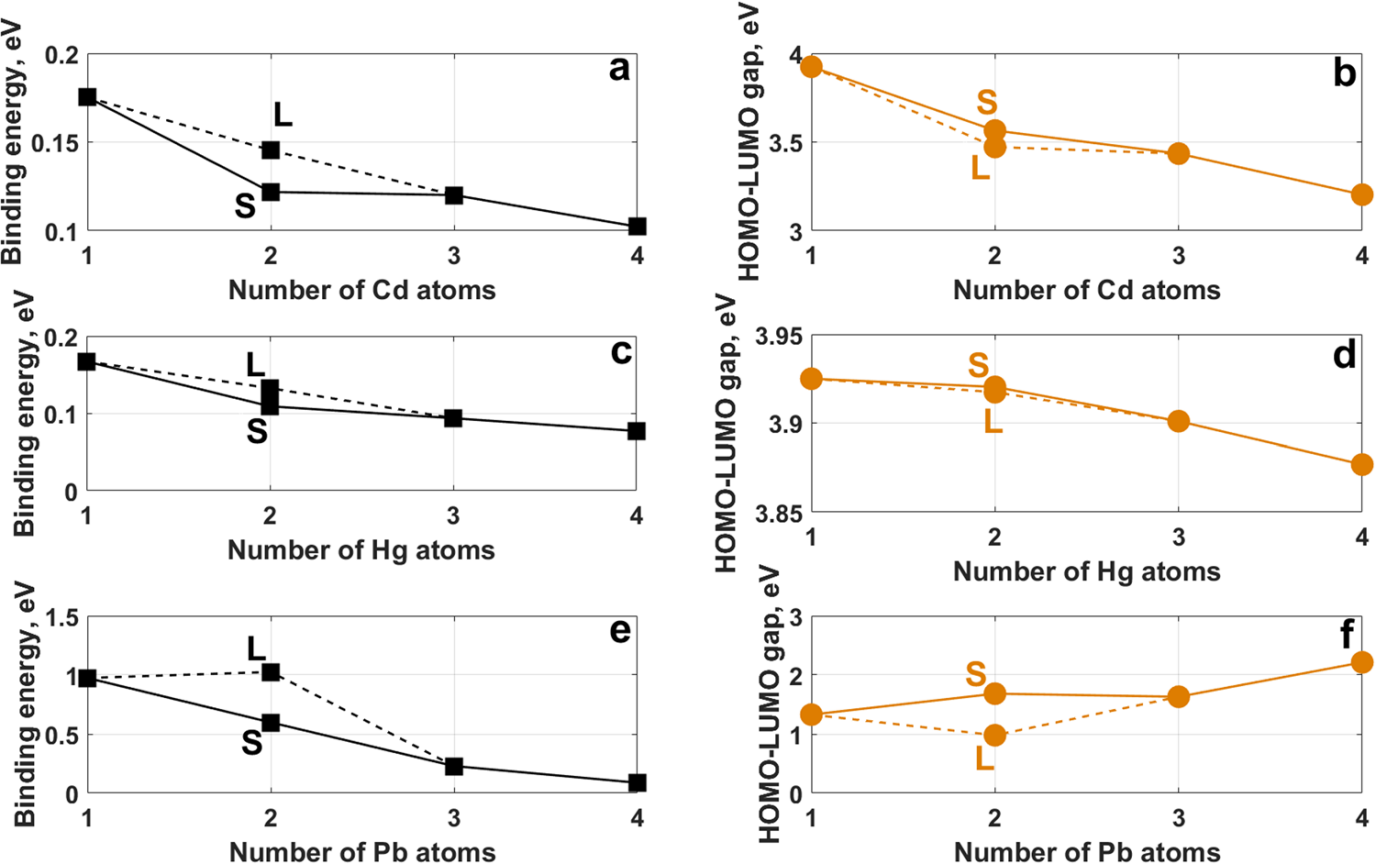
Dependences of the binding energy and HOMO-LUMO gap on the number of heavy metals’ atoms on the C_16_H_10_ GQDs: Cd (**a,b**), Hg (**c,d**), and Pb (**e,f**), respectively. L and S denote the longer and shorter initial distances between adsorbed adatoms on graphene surface.

Figure 9 shows how the binding energies of metallic clusters adsorbed on C_16_H_10_ GQDs vary with the number of atoms in the cluster. The binding energy of an isolated neutral Cd and Hg atoms on the graphene quantum dot is 175 meV and 167 meV, respectively, but decreases upon increasing the number of adsorbed atoms (from 1 atom to 4 atoms) to 102 meV and 76 meV. This decrease is caused by the competition between metal-surface and metal-metal interactions. For the case of shorter metal-metal distances the binding energy becomes lower with an increasing number of adatoms. Increasing the number of Pb atoms adsorbed on the graphene surface results in an even more dramatic decrease in the binding energy. This is driven by the energetic preference of Pb atoms to cluster when adsorbed on graphene.

The HOMO-LUMO gap of the combined system is also dependent on the number of adatoms. Figure 9 shows that the HOMO-LUMO gap is weakly dependent on the size of the Hg cluster, but is much more strongly dependent on the size of Pb and Cd clusters. As expected, due to the highest binding energy of the Pb cluster, there is a significant reduction in the magnitude of the HOMO-LUMO gap compared to that of the pristine graphene quantum dot.

### Substitutional defects

#### Structural and electronic properties

**Figure 10:**
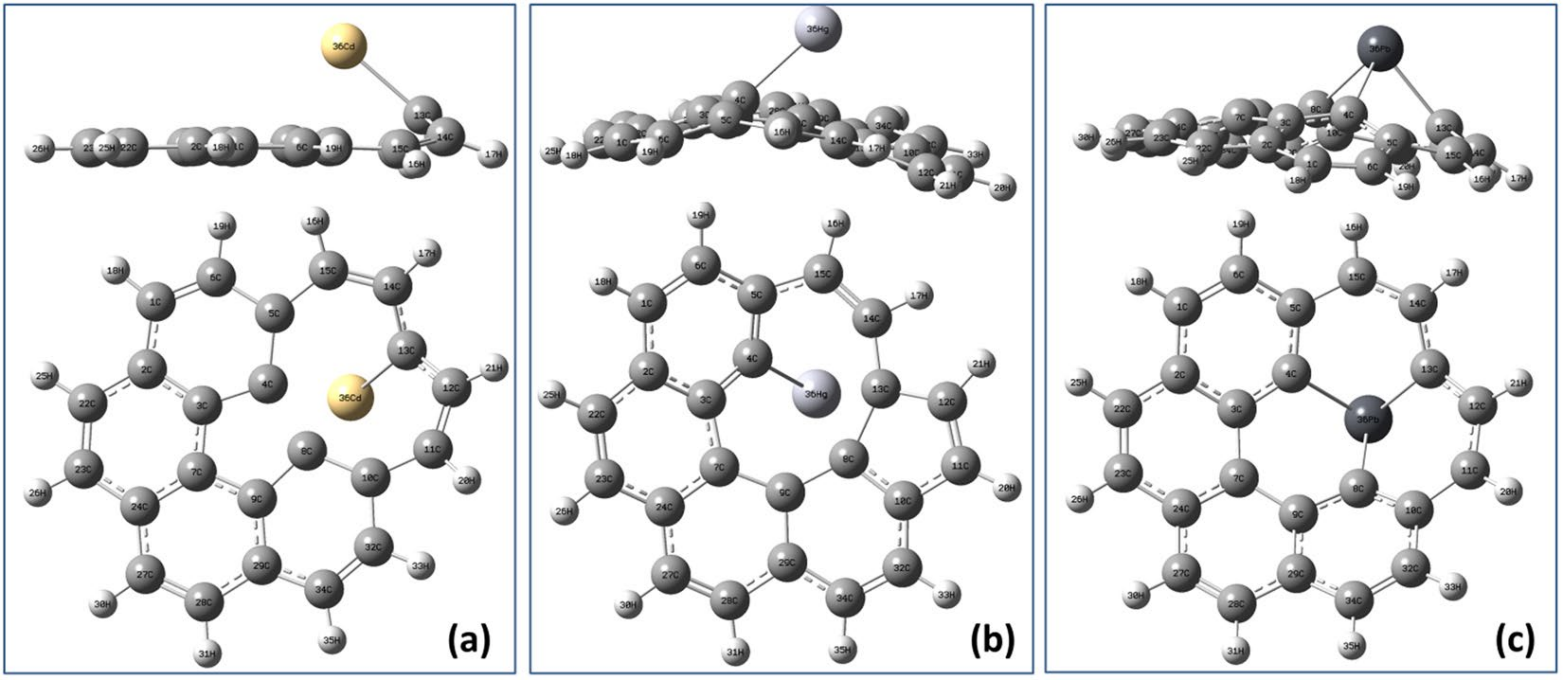
Optimized structure of a C_23_H_12_ graphene quantum dot with a substitutional (**a**) Cd, (**b**) Hg and (**c**) Pb defect.

Substitutional and vacancy defects in graphene can occur during the growth stage and may significantly modify the typical electronic and vibrational fingerprints of graphene. Heavy metal atoms may therefore also adsorb on a vacancy site as well as on a defect-free carbon surface and it is important to distinguish these two events. In this section, we investigate how the substitutional replacement of a carbon atom in the GQD with a heavy metal atom affects the local structural disorder and changes the electronic and Raman spectra of C_24_H_12_ quantum dots. A carbon atom belonging to the central hexagonal ring is replaced with a heavy metal atom. The relaxed structures after geometry optimization are shown in Fig. 10. Due to the fact that the covalent radii of the heavy metal atoms are significantly larger than that of carbon (75 pm) at 146 pm, 132 pm and 144 pm for Cd, Hg and Pb, respectively, the metal atoms are ejected from the *sp* ^2^ plane. The Cd-C, Hg-C and Pb-C bonds are longer than the C-C bond at 2.22 Å, 2.28 Å and 2.26 Å, respectively. As a result, the graphene quantum dot experiences a considerable distortion. We define the out-of-plane distortion of the GQD as:

1 $${\rm{\Delta }}h={Z}_{max}-{Z}_{ave}$$

where *Z*_*max*_ is the maximum value of the out-of-plane coordinates of the carbon atoms and *Z*_ave_ is the average value. We find that Δ*h* due to the Cd doping (0.912 Å) is much lower than that due to Hg and Pb doping (1.779 and 1.568 Å, respectively). This difference may be understood in terms of electron affinity: Hg has the lowest value of the electron affinity among the heavy metals considered here. A lower value of electron affinity results in lower values of charge transfer and binding energy, implying that the mercury atom does not accept electrons as easily. For this reason the C-Hg bond length is longer than the C-Cd and C-Pb bond lengths and the remaining carbon atoms are reorganized into a distorted graphene quantum dot. For the case of a substituting Pb atom, the graphene quantum dot is also distorted, but now Pb is bonded to three carbon atoms, due to its high electron affinity.

The charge on the substituting heavy metal atoms is changed significantly compared to the charge on the isolated atoms. The charges on Cd, Hg and Pb atoms are calculated to be + 0.843e, + 0.650e and + 0.544e, respectively.

**Figure 11:**
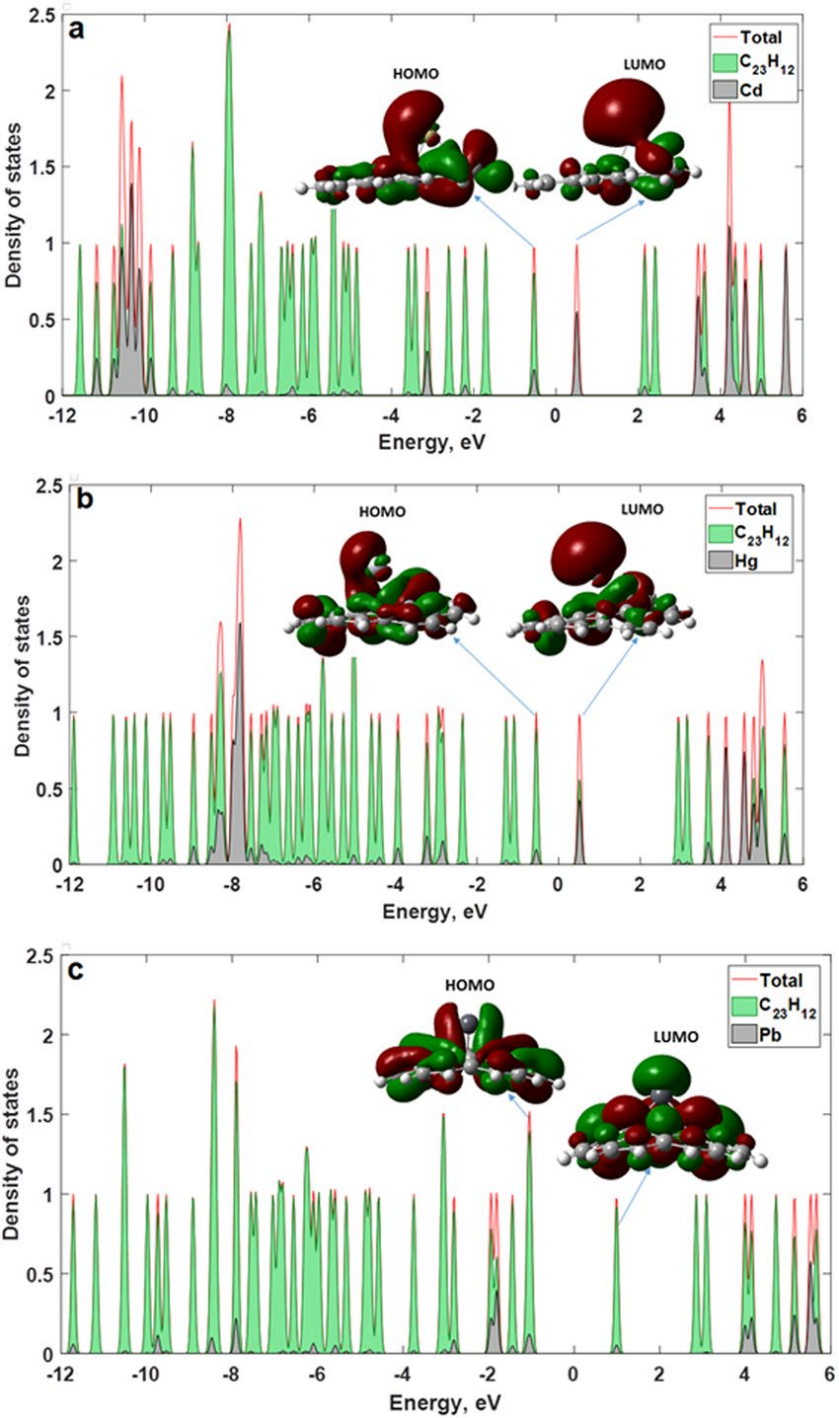
Total and projected DOS (PDOS) for graphene quantum dots with atoms of the heavy metals, which substitute the carbon ones in planar structure: C_23_H_12_ cluster with Cd (**a**), C_23_H_12_ cluster with Hg (**b**) and C_23_H_12_ cluster with Pb (**c**). Insets represent corresponding iso-surfaces of the molecular orbitals. The Fermi level is set to be at zero (midgap) by defining E_F_ = (E_HOMO_ + E_LUMO_)/2.

Furthermore, the interaction between the metal atoms and the graphene quantum dot has a significant influence on the electronic properties of the coronene system. The total and projected DOS of the interacting systems are shown in Fig. 11. For the case of the Cd-C_23_H_12_ GQD (Fig. 11a), the primary contribution to the LUMO is from the Cd atom, whereas the orbitals of the C_23_H_12_ dominate the HOMO (Fig. 11a). For the Hg-C_23_H_12_ GQD, one can observe that the contribution of the Hg atom to the formation of the bottom of the conduction band is slightly less than the contribution of orbitals from C_23_H_12_ (Fig. 11b). In the case of Pb-doped cluster, the orbital mixing at the top of the valence band and at the bottom of the conduction band is weak and Pb contribution to the HOMO and LUMO is negligible (Fig. 11c). A minimal hybridization of the Pb-involving orbitals with C_23_H_12_ molecular orbitals is not fully understood, but we suppose that it may be associated with the fact the substituting Pb atom loses their metallic characteristics. Due to the bonds formed with three C atoms, the electron-density distribution is thus more uniform, leading to an enhanced HOMO-LUMO gap. To better understand the electronic structure of interacting cluster, we also show the frontier molecular orbitals (see Inserts in Fig. 11). For the case of pristine graphene, the LUMO and HOMO are delocalized over the entire surface of the coronene cluster. At the same time, LUMO orbitals of the Cd-C_23_H_12_ are preferentially localized at the cadmium atom, whereas the HOMO is shared by both the Cd adsorbate and the GQD. The distribution of the HOMO and LUMO for Hg-doped cluster is roughly the same as in the case of the Cd-doped cluster. Due to the formation of three equivalent C-Pb bonds after interaction between the GQD with Pb, the more homogeneous electron density distribution occurs and thus LUMO and HOMO are delocalized over Pb-C_23_H_12_.

Features of electron density distribution that depend on the interaction of the coronene with heavy metals are correlated with the values of the HOMO-LUMO gap. The size of the HOMO-LUMO gap follows the sequence C_23_H_12_ > Pb-C_23_H_12_ > Hg-C_23_H_12_ > Cd-C_23_H_12_. This trend is accompanied by changes in degree of delocalization. The smallest values of the HOMO-LUMO gap (~1.04 eV and 1.06 eV) is observed for Cd-doped and Hg-doped GQDs due to the strong localization of the LUMO orbitals on the Cd and Hg atoms. The HOMO-LUMO gap of Pb-C_23_H_12_ is 2.01 eV due to the weak contribution of the Pb to the total DOS.

#### Vibrational properties

**Figure 12:**
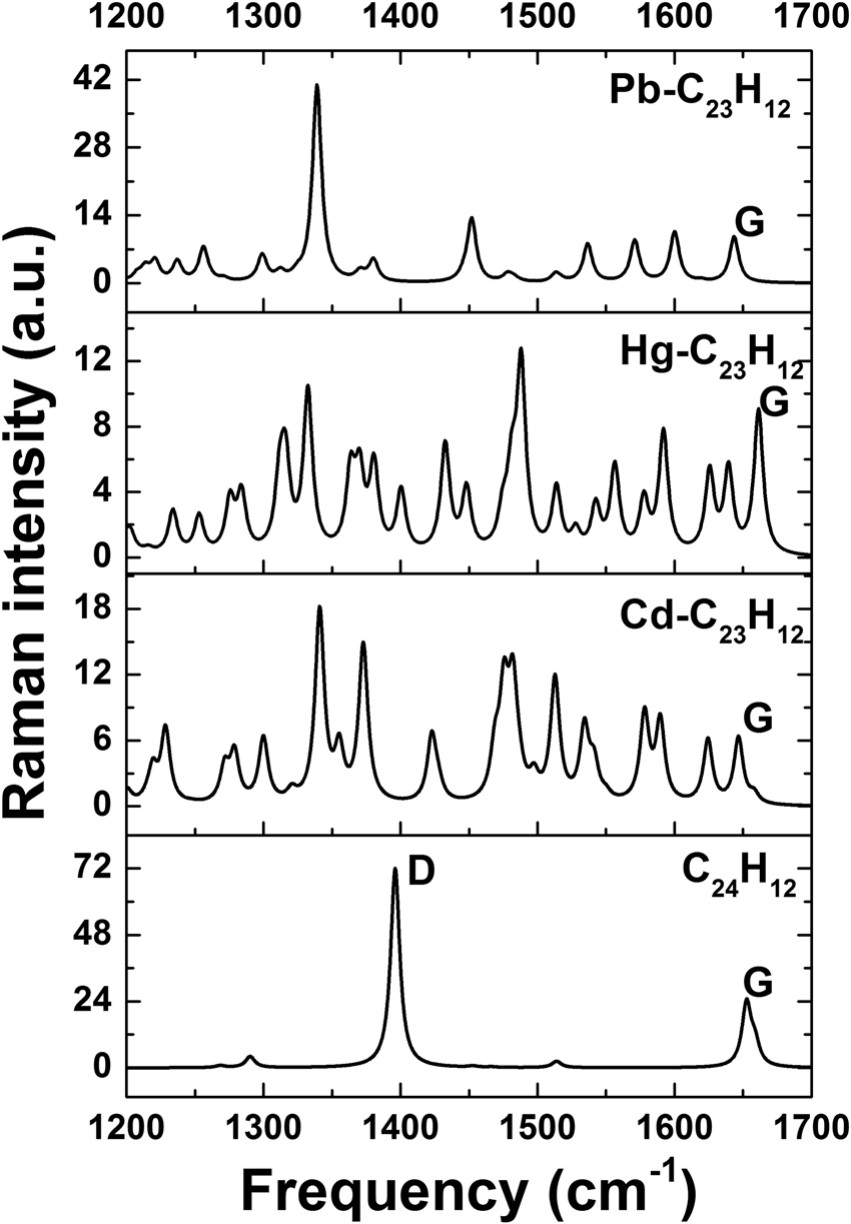
Raman spectra of the defective C_24_H_12_ graphene quantum dots with and without different atoms of heavy metals.

Finally, we discuss the vibrational properties of the defective GQDs. The substitution of a carbon atom with a heavy metal atom will significantly influence the out-of-plane and in-plane movement of the host GQD, leading to a modification of the intensities and frequencies of the allowed phonon modes. The Raman scattering spectra were calculated for pristine coronene and for the systems with heavy metal adsorbants. Due to the hydrogen-terminated edges of the GQDs, there will not only carbon-related phonon modes, but also mixed modes corresponding to C-H stretching and vibrations involving only hydrogen atoms. We consider the Raman spectra located in the frequency region from 1200 cm^−1^ to 1700 cm^−1^, as it includes the characteristic fingerprint of in-plane C-C movements, that is, the *G*-mode. It is generally accepted that the relationship between the *D* and *G* modes can be regarded as an effective parameter for quantifying disorder in graphene, which is induced by point defects, external doping and edges (crystallite borders, hydrogen termination etc.)^60^. We do not discuss here the modes up to 1200 cm^−1^ (C-C out-of-plane, C-H in-plane and out-of-plane vibrations) and hydrogen-related high-frequency vibrations above 1700 cm^−1^. In addition, it is important to note that the local phonon modes induced by substitution manifest themselves only in low-frequency region of the Raman spectra due to very large value of oscillator mass and the so-called isotopic effect. Due to the *D*_6H_ symmetry and the structural properties of C_24_H_12_ cluster, the irreducible representation and selection rules imply that there are 24 Raman active modes: 6*A*_1g_, 6*E*_1g_, and 12*E*_2g_^61^. As can be seen in Fig. 12, the Raman spectrum of the pristine coronene is dominated by two prominent peaks at 1396 cm^−1^ and 1652 cm^−1^. These features can be assigned to *A*_1g_ and *E*_2g_ phonons, in good agreement with ref. 62 For the case of infinite ideal graphene, only the *E*_2g_ zone center phonon is allowed by the selection rules and therefore only the *G* mode is usually observed in the Raman spectra of the high-quality graphene. The activation of the *A*_1*g*_ zone-boundary mode (equivalent to the *D* band) is associated with the relaxation of the *k* = 0 Raman selection rule due to disorder in the graphene sheet^63^. In the case of the graphene quantum dot, the edges are responsible for the presence of this zone-boundary phonon mode. Furthermore, we can see that the intensity of the *D* band is higher than that of the *G* band, indicating the important role of the hydrogen termination in the Raman scattering processes in small GQDs.

The substitution of a C atom with a heavy metal atom causes a break the translational symmetry of the GQDs and therefore the relaxation of the momentum conservation required for activating other zone-boundary phonons. In this case, the intensity of the normal *G* mode for defective coronene is reduced compared to pristine coronene. A set of new Raman peaks corresponding to in-plane carbon vibrations can be observed due to the presence of Cd, Hg and Pb (Fig. 12). In addition, we noticed the significant shift of the *G* mode towards lower frequency for Cd-C_23_H_12_ and Pb-C_23_H_12_ clusters and towards higher frequency for Hg-C_23_H_12_ cluster. This trend is associated with the doping-induced oscillatory behavior of the phonon *G*-mode frequency. These changes can be related to the significant changes in local bonding environment due to the substitution of the carbon atoms with HM atoms. As a result, the atoms vibrate in a localized mode. Therefore, we observe not only the unique Raman peaks associated with the dopant, but also a shift in the *G*-peak. Each of the three heavy metals causes a unique local disorder, and thereby a unique Raman fingerprint.

### Optical properties: effect of the edge geometry and size of GQD on UV-vis adsorption spectra

The electronic and optical properties of graphene quantum dots depend strongly on their shape, size and edge disorder^6^. GQDs can be divided into two types depending on their edge architecture: armchair and zigzag. We now consider how variations in both terminations and size influence the binding energy, the HOMO-LUMO gap, the charge transfer and the UV-visible adsorption spectra of the graphene quantum dots after complexation with HMs. We focus mainly on the understanding of the fundamental correlation between the physical nature of the excited electronic transitions in *sp* ^2^ conjugated structure of GQDs and the size-/edge-dependent strength of interaction between HMs and GQD.

**Figure 13:**
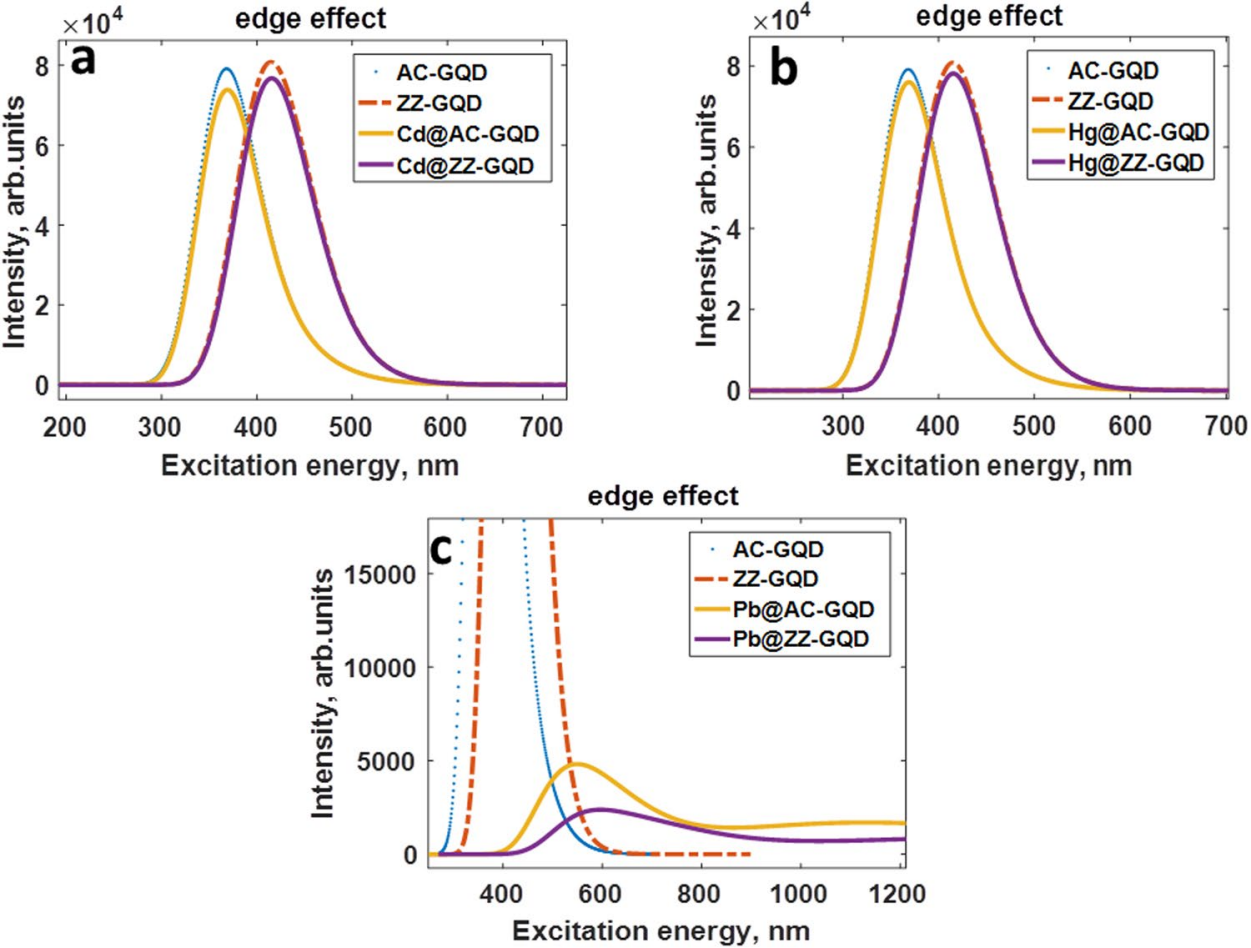
UV/Vis absorption spectra of the zigzag-edged and armchair GQDs before and after complexation with neutral heavy metal adatoms, namely (**a**) Cd, (**b**) Hg and (**c**) Pb.

For this aim, we have explored the edge and size effects by optimizing the structures of zigzag-edged and armchair-edged GQDs with different diameters before and after applying heavy metals (Tables S1–S3 represent all structures investigated, Supplementary materials). It was revealed that the pristine GQDs with zigzag edges have a reduced HOMO-LUMO gap in comparison to those with armchair edges (Fig. S1, Supplementary materials). This can be explained by the presence of the localized states on zigzag edges, which is in good agreement with previous investigations^6^. Furthermore, the energy gap of the zigzag-edged GQDs decreases more rapidly with increasing the size of the GQD than the HOMO-LUMO gap of the armchair-edged GQDs (Fig. S1, Supplementary materials). This difference can be clearly seen in UV-vis adsorption spectra of the C_54_H_22_ (with armchair edges) and C_54_H_18_ (with zigzag edges) GQDs, Fig. 13a. Both of them exhibit strong absorption peaks in the optical region, which can be attributed to π-π* transitions related to the presence of C = C bonds. These transitions correspond to the lowest singlet excited state (having the B_u_ symmetry). Compared with the armchair GQDs, the absorption peak of zigzag GQDs exhibits a red-shift of 45 nm, while the oscillator strength of dominating transitions are similar in both cases. The adsorption spectra of the armchair GQDs is mainly dominated by two peaks at 371 and 369 nm. The former is associated with H-1- > L + 1 (75%) and HOMO- > LUMO (16%) electronic transitions and the latter is originated from H-1- > LUMO (46%) and HOMO- > L + 1 (48%) transitions. The same electronic transitions contribute to the adsorption spectra of the zigzag-edged GQDs.

When adsorbed by nanographene surface, heavy metal species introduce new local states (molecular orbitals) into the electronic structure of graphene nanomaterials, thereby modifying the total density of states as well as optical properties, which are strongly governed by electronic structure. As a result, a new kind of the electronic transitions (singlet excited states with *A*_g_ symmetry), namely *n*-π* transitions can occur. These electronic transitions can be regarded as unique electronic signatures serving for detection of the heavy metals.

In virtue of the physisorption nature of the interaction between neutral Cd and Hg adatoms with GQDs, the adsorption spectra of the Cd@GQD and Hg@GQD remain unchanged and just slightly change intensities compared to the pristine zigzag and armchair graphene quantum dots (Fig. 13a and b). As was discussed before the binding energy of the Cd adatom is slightly larger than that of Hg adatom and thus more Cd-related electronic states are expected to be involved into the optical transitions. In particular, it is evidenced by the fact that two distinct adsorption peaks at the 370 nm and 372 nm of the Cd@GQD are dominated by H-2- > LUMO and H-2- > L + 1 transitions, whereas H-2 orbitals are not involved in optical transitions in the case of the Hg@GQDs.

The adsorption of neutral Cd and Hg adatoms is slightly dependent on the size of the GQD and the type of the edge termination. Analyzing the geometrical configuration of the neutral Cd and Hg at the surface of the varying sized zigzag and armchair edged GQDs, it was revealed that in most cases the hollow site is the most favorable position for Cd and Hg adsorption. Our calculations show that the binding energy increases with increasing diameter of GQD, reaching the saturation level for larger quantum dots (Fig. S2, Supplementary Materials). This increase is primarily due to the size-dependent increase in charge transfer from Cd (Hg) to GQD, which enhances the interaction between the metal and *sp* ^2^ carbon surface. As is clear from the Fig. S3 (Supplementary Materials), the transferred charge monotonically increases, reaching the maximal magnitude of electron donation for armchair edged C_60_H_26_. The electron donation reaches 0.08e^−^ and 0.09e^−^ for Cd and Hg adatoms, respectively. The charge transfer vanishes as the GQD size decreases and changes direction for Metal-C_6_H_6_ system. At the same time, the binding energy of neutral adatoms adsorbed on the surface of the large armchair-edged GQDs is larger than that of zigzag-edged GQDs. Due to the slight non-covalent interaction between cadmium (mercury) and graphene, the HOMO-LUMO gap of zigzag and armchair Cd@GQDs and Hg@GQDs follows the size dependence of the energy gap of their pristine counterparts. Taking these features into account, it is reasonable to assume that UV-vis adsorption spectra of the GQDs after complexation with Cd and Hg are not strongly affected by incoming adsorbates and no significant red-shift is expected.

In contrast, the neutral Pb adatoms interact more strongly with GQDs, always occupying the bridge site between two nearest carbon atoms for all considered sizes. In this case, there is no clear dependence of the binding energy, the charge transfer and the HOMO-LUMO gap of the Pb@GQDs on the size of the GQD, as has been demonstrated in Table S4 and S5 (Supplementary materials). Binding energy and charge transfer did not demonstrate a systematic monotonic trend as a function of the size of the GQD. Donor-like Pb adatom interacts with each GQDs in unique manner, but as was expected the armchair GQDs provide the conditions for stronger interaction. Interestingly that the adsorption ability of several small graphene quantum dots to Lead exposure is even higher compared to large nano-objects. It is apparent that in the case of smaller objects the edges of the GQDs can play a role of more effective active adsorption sites. Such a strong interaction implies the *n*-type doping of GQDs and thus a red shift in the absorption spectra is expected. Indeed, UV-visible spectrum of armchair-edged graphene quantum dot after complexation with neutral lead atom showed two strong absorption peaks around 512 nm and 552 nm which are mainly attributed to H-1- > LUMO and HOMO- > L + 9 transitions, respectively (Fig. 13c). The absorption maxima at 555 nm, 571 nm and 729 nm corresponding to H-1- > LUMO, HOMO- > L + 9 and HOMO- > L + 6 transitions, respectively, are observed on the UV-vis spectrum of the zigzag-edged Pb@GQD. Adsorption of a single Pb atom yields distortion of GQDs and, as a result, the geometric symmetry-breaking induced by the interaction with Pb adatoms leads to decrease in oscillator strengths of the electronic transitions. For this reason, the intensity of the red-shifted adsorption peaks of the Pb@GQDs is reduced compared to that of the pristine quantum dots.

**Figure 14:**
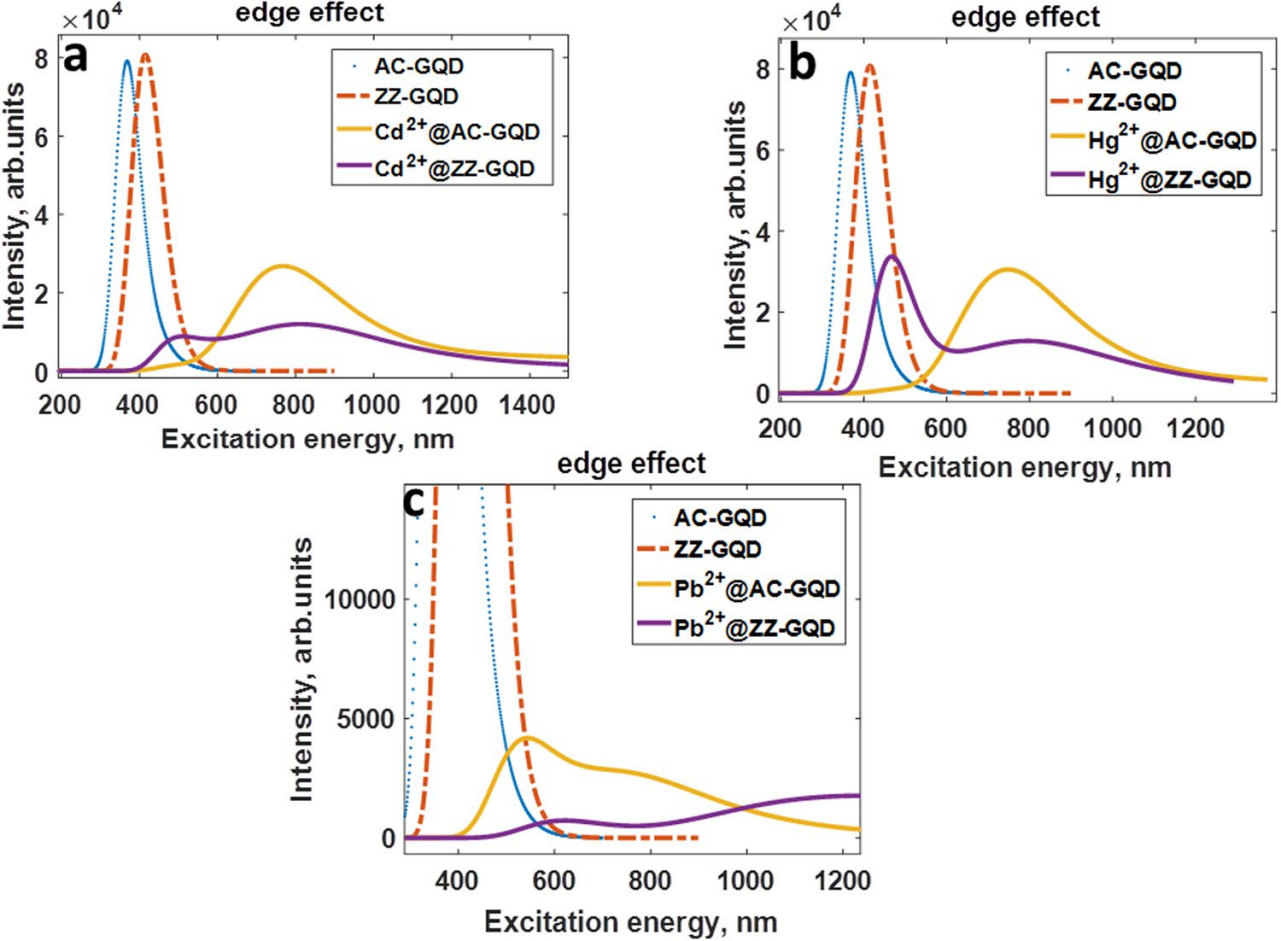
UV/Vis absorption spectra of the zigzag-edged and armchair-edged GQDs before and after complexation with divalent heavy metal ions, namely (**a**) Cd^2+^, (**b**) Hg^2+^ and (**c**) Pb^2+^.

Analyzing the adsorption spectra of the graphene quantum dot systems after complexation with divalent heavy metal ions (Fig. 14a–c), we revealed the huge red-shift and quenching of the adsorption peaks compared to those of the pristine graphene quantum dots. Table S6 (Supplementary Materials) systemizes the electronic transitions, which are responsible for the adsorption peaks. This effect can be understood in terms of doping effect. Indeed, the chemisorption of the divalent ions implies the strong *p*-doping in graphene and, as a consequence, modulation of the electronic properties.

**Figure 15:**
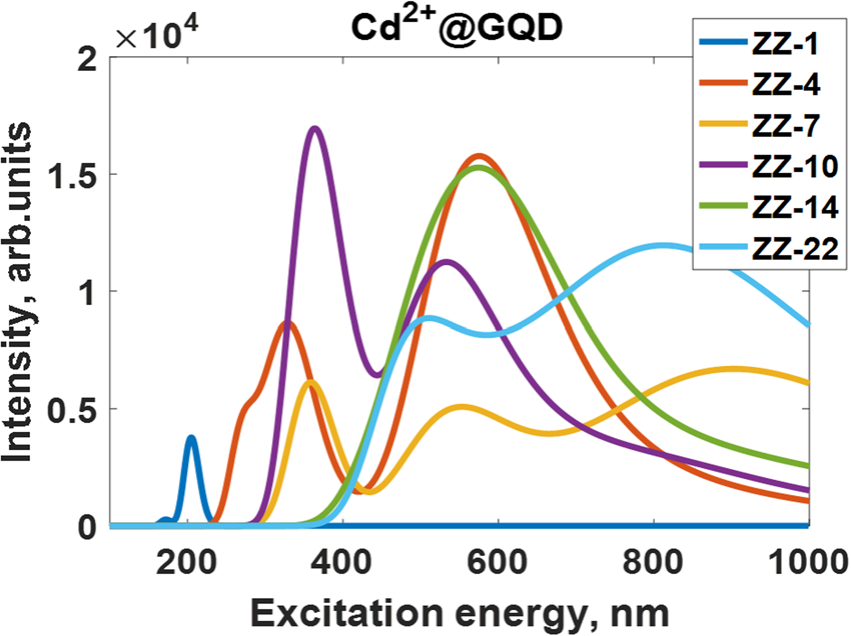
UV/Vis absorption spectra of the zigzag-edged GQDs after complexation with divalent Cd^2+^ ions depending on the size of the GQDs. ZZ denotes a kind of edge, while the following integer corresponds to the number of hexagonal rings in GQD.

In fact, the heavy metal ions on nanographene surface act as electron acceptors. Because of this, a drop in the HOMO is observed and thus adsorption spectra of the GQDs are significantly changed. It should be mentioned that the binding energy of HM ions and the charge transfer from GQDs to HMs ions are highly sensitive to the size of the GQD. As one can see from the Figs S4 and S5 (Supplementary materials), the binding energy of divalent metal ions gradually increases with increasing the size of GQD. At the same time, HM ions can accept more and more electrons in the case of large GQDs (Fig. S5, Supplementary materials), thereby more strongly affecting the adsorption spectra. It is clearly evidenced by the monotonic decrease of the magnitude of Mulliken charge on Cd^2+^, Hg^2+^ and Pb^2+^ adsorbed on the surface of zigzag-edged and armchair-edged graphene quantum dots (Fig. S5, Supplementary materials). Figure 15 demonstrates the UV-vis spectra of the zigzag-edged GQDs after complexation with Cd^2+^ divalent ions depending on the size of the GQD. It is clearly seen that increasing the charge transfer and binding energy with increasing the size leads to a red-shift of the adsorption peak and an appearance of new singularities, which are originated from the electronic transitions involving Cd-related electronic states. This trend is in line with the size-dependence of the HOMO-LUMO gap (Fig. S6). Effect of doping on HOMO-LUMO gap, is comparatively, more significant in the case of the Hg^2+^@GQD compared to Cd^2+^@GQD and Pb^2+^@GQD.

We anticipate that the observed effects should stimulate the development of novel devices for sensing applications. For an example, such applications could be based on the sensitive nano-sized flakes of graphene quantum dots dispersed in solvents. The fundamental working principle is associated with (i) interaction of heavy metals with sensitive GQDs and (ii) subsequent measurements of the optical response of graphene-based solutions to exposure of the heavy metal species. Since the optical properties of graphene nanomaterials are significantly affected by incoming adsorbates, it is expected that each of HMs will lead to unique optical fingerprint. In fact, collecting the information about the peak dispositions, peak quenching and novel electronic singularities gives us a possibility of a deeper understand of the heavy metals effect on the properties of the graphene-related materials. Thus, a comprehensive analysis of the optical response of the graphene-based sensing platform to HMs exposure can lead to an effective tool for a fast detection of the heavy metals in aqueous solutions and even in biological liquids.

## Summary and Concluding remarks

DFT calculations were employed to investigate the interaction between neutral and charged heavy metal atoms of cadmium, lead and mercury and different zigzag and armchair-edged GQDs. The binding energy, the charge transfer and the equilibrium distances between the adsorbates and graphene quantum dots are found to be strongly dependent on the adsorption site, the charge state of the metal adsorbate, the size/edge geometry of the GQDs and the concentration of heavy metals on graphene quantum dot. We find that the neutral atom of Pb tends to bind more strongly to GQD than either Cd or Hg, acting an electron donor. This can be explained by differences in their ionization energies. We show that the most favorable adsorption site for Cd and Hg is the high-symmetry hollow site, whereas Pb binds preferentially on the bridge site. The adsorption properties of the charged ions differ significantly from those of the neutral atoms, due to the chemisorption nature of the interaction. The charged heavy metal species were found to act as electron acceptors in the GQDs–HMs systems. A general increase of the binding energy of the neutral and charged heavy metals species, and the corresponding charge transfer increase, with the GQD size was found. The adsorption ability of the armchair GQDs to HMs is stronger compared to zigzag GQDs. The energetic barriers for Cd or Hg motion across the GQDs are very small. The Pb atom migrates mostly along the C-C bond, avoiding the energetically unfavorable hollow site. The binding energy of heavy metals decreases with an increasing a number of metal adatoms. Such behavior is driven by the energy gain originating from the competition between metal-metal interaction and metal-GQD complexation. We find that unlike the formation of a Pb dimer on graphene quantum dots, the formation of Hg and Cd dimers is not energetically favorable. We also show that the substitution of a carbon atom in the GQD by the heavy metal atoms causes a significant distortion of the nanographene plane, accompanied by the protrusion of the metal atoms from the *sp* ^2^ surface. We showed that the Raman scattering spectra of the GQDs provides a unique fingerprint of each interacting system. In view of the high sensitivity of the vibrational properties of graphene quantum dots to adsorbates, graphene quantum dots-based Raman sensors can be used to detect toxic heavy metals.

Finally, using the results of TDDFT calculations we explained in detail the nature of excited states in the zigzag and armchair GQDs before and after complexation with HMs. Based on the results, we discussed how UV-vis adsorption spectra can be used for development of realistic optical sensors. Our study revealed that due to the weak interaction of the neutral Cd and Hg adatoms with GQDs, the adsorption spectroscopy is not appropriate method for detection of these two. On the other hand, a probing of the GQDs with substitutional defects by Raman spectroscopy can provide more valuable information about Cd^0^ (Hg^0^)-GQDs interaction. The adsorption spectra of the GQDs after complexation with heavy metal ions are found to be highly sensitive to the size and edge configuration of the GQD. In this case, the doping effect induces a significant red-shift of the absorption spectra of both zigzag and armchair GQDs as well as an appearance of new adsorption peaks in long wavelengths region. Our results show that the effect of the chemisorbed HM ions on UV-vis absorption spectra of GQDs is very complex and the changes in the nature of the excited electronic transitions correlate directly with charge transfer from electron-donating GQDs to acceptor-like heavy metal ions. The obtained results provide insightful nano-engineering approach based on size and edge geometry control toward development of advanced optical graphene-based sensors for detection of the heavy metals in different oxidative states.

## Methods

In this paper, DFT calculations were performed using the Gaussian 09 package^64^, to investigate the interaction of GQDs with three different heavy metal atoms, namely cadmium, mercury and lead. Geometry optimization of the interacting systems was done at the Becke3LYP level of DFT with a 6–31 G basis set for carbon and a basis set developed by the Stuttgart-Dresden-Bonn group for the heavy metal atoms^65^, using the default convergence criteria. We use C_16_H_10_, C_24_H_12_, C_30_H_16_, and C_54_H_18_ as representatives of GQDs.

We also determined how these heavy metal adsorbants interact with extended graphene. This was done using the PAW^66^ method as implemented in the Vienna ab initio simulation package^67, 68^ (vasp-5.3), which utilizes periodic boundary conditions. In this case, infinite graphene was described using a 4 × 4 supercell, i.e., with 32 carbon atoms. The Perdew-Burke-Ernzerhof (PBE)^69^ generalized gradient approximation (GGA) was employed. The plane wave basis set was converged using an 800 eV energy cutoff. Structural relaxations were carried out using a 5 × 5 × 1 *k*-point Monkhorst-Pack mesh^70^ to sample the Brillouin zone. A 9 × 9 × 1 mesh was then used to determine the total energies. The DFT-D3 method of Grimme was used to describe van der Waals interactions^71^. A vacuum layer of at least 20 Å was included in the direction normal to the surface to ensure no spurious interactions between repeating slabs. The positions of the carbon atoms, as well as the adatom, were optimized until all residual forces were less than 0.01 eV·Å^−1^.

The binding energy of a heavy metal adatom to a graphene quantum dot is defined as:

2 $${E}_{b}=-[{E}_{GQD+HM}-({E}_{GQD}+n{E}_{HM})]/n$$

where ${E}_{GQD+HM}$ is the total energy of the interacting graphene quantum dot-heavy metal system, ${E}_{GQD}$ is the total energy of the isolated graphene quantum dot, ${E}_{HM}$ is the total energy of an individual heavy metal atom or ion and *n* is the total number of interacting heavy metal atoms or ions. In a case of the interaction between graphene quantum dots and metal clusters (for example, dimers) we cannot neglect the contribution of the metal-metal attraction to binding energy of the heavy metal and thus the later can be evaluated from the energy difference between the total potential energy of the interacting “GQD-metal cluster” system, ${E}_{GQD+MC}$, and the sum of the total energies of graphene QD, $\,{E}_{GQD}$, and metal cluster, $\,{E}_{MC}$:

3 $$\,{E}_{b}=-[{E}_{GQD+MC}-({E}_{GQD}+{E}_{MC})]$$

It should be mentioned that the binding energies have corrected for the basis set superposition error (BSSE) by means of counterpoise method^72^.

The excited electron states – transitions between occupied and unoccupied states – of the zigzag and armchair varying sized GQDs before and after applying the heavy metals species were investigated by means of the time-dependent density functional theory (TD-DFT) approach. 100 single excited states were involved to calculate the ultraviolet-visible adsorption spectra at the TDDFT/B3LYP level of theory using 6–31 basis set, implemented in the GAUSSIAN 98 program. The oscillator strengths for each transitions is proportional to the intensities of absorption peaks. The shift and quenching the adsorption peaks is due to the charge transfer between graphene quantum dots and heavy metal species.

## Electronic supplementary material

Supplementary Materials
