# Supplementary material for: On the interaction of toxic Heavy Metals (Cd, Hg, Pb) with graphene quantum dots and infinite graphene
Source: Sci Rep. 2017 Jun 21;7:3934. doi: 10.1038/s41598-017-04339-8 (PMC5479795; doi:10.1038/s41598-017-04339-8)
Supplement: Supplementary file 1 — Supplementary Materials [file 41598_2017_4339_MOESM1_ESM.pdf]

**SUPPLEMENTARY INFORMATION**

# On the interaction of toxic Heavy Metals (Cd, Hg, Pb) with graphene quantum dots and infinite graphene

Ivan Shtepliuk[1], Nuala M. Caffrey[1,2], Tihomir Iakimov[1], Volodymyr Khranovskyy[1], Igor A. Abrikosov[1,3] and Rositsa Yakimova[1]

[1]*Department of Physics, Chemistry and Biology, Linköping University, SE-58183, Linköping, Sweden*

[2]*School of Physics and CRANN, Trinity College, Dublin 2, Ireland*

[3]*Materials Modeling and Development Laboratory, National University of Science and Technology "MISIS", Moscow, Russia*

*Corresponding author: Ivan Shtepliuk, e-mail: ivan.shtepliuk@liu.se*

**Table S1. Structures of zigzag edged and armchair-edged GQDs.** "ZZ" and "AC" denotes a kind of edge, while the following integer corresponds to the number of hexagonal rings in GQD.

| **Zigzag Graphene Quantum Dots** | **Structure** | **Armchair Graphene Quantum Dots** | **Structure** |
|---|---|---|---|
| Benzene $C_6H_6$<br>ZZ-1 | 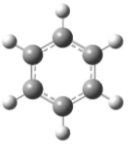 | Triphenylene $C_{18}H_{12}$<br>AC-4 | 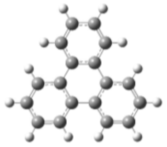 |
| $C_{13}H_9$<br>ZZ-3 | 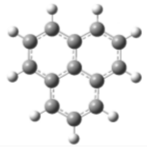 | $C_{23}H_{14}$<br>AC-6 | 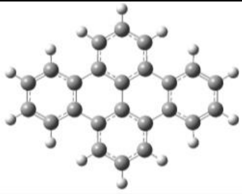 |
| Pyrene $C_{16}H_{10}$<br>ZZ-4 | 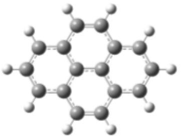 | Tribenzo (b,n,pqr)perylene<br>$C_{30}H_{16}$<br>AC-8 | 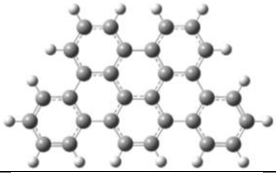 |
| Coronene $C_{24}H_{12}$<br>ZZ-7 | 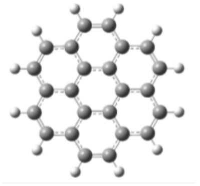 | Tribenzo(A,D,G)Coronene<br>$C_{36}H_{18}$<br>AC-10 | 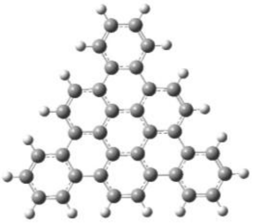 |
| Ovalene $C_{32}H_{14}$<br>ZZ-10 | 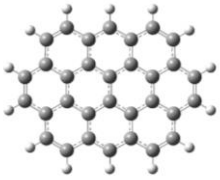 | HexabenzoCoronene<br>$C_{48}H_{24}$<br>AC-13 | 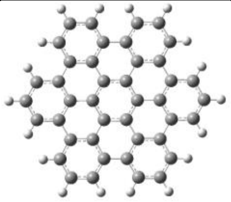 |
| Circumpyrene $C_{42}H_{16}$<br>ZZ-14 | 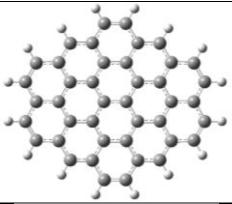 | $C_{54}H_{22}$<br>AC-17 | 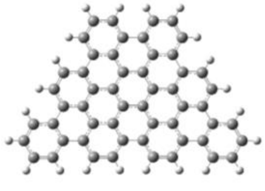 |
| Circumcoronene<br>$C_{54}H_{18}$<br>ZZ-22 | 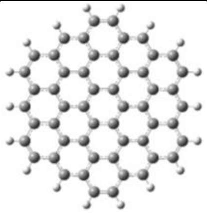 | $C_{60}H_{26}$<br>AC-19 | 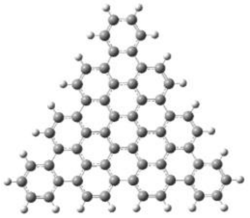 |

**Table S2. Structures of zigzag edged and armchair-edged GQDs interacting with neutral heavy metal species.** "ZZ" and "AC" denotes a kind of edge, while the following integer corresponds to the number of hexagonal rings in GQD.

| ZZ-GQD | Structure | | | AC-GQD | Structure | | |
|---|---|---|---|---|---|---|---|
| | Cd | Hg | Pb | | Cd | Hg | Pb |
| ZZ-1 | 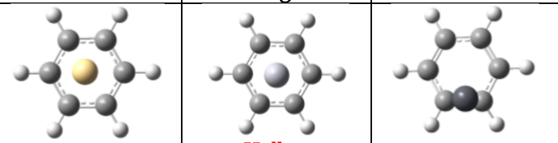 Hollow | Hollow | Bridge | AC-4 | 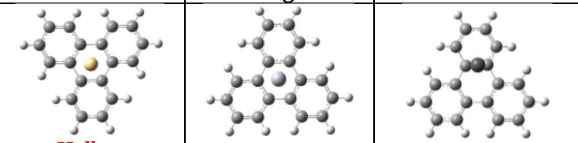 Hollow | Hollow | Bridge |
| ZZ-3 | 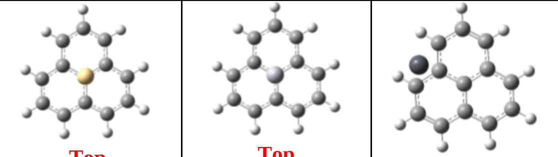 Top | Top | Bridge | AC-6 | 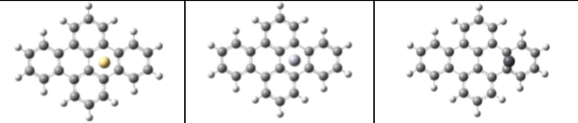 Hollow | Hollow | Bridge |
| ZZ-4 | 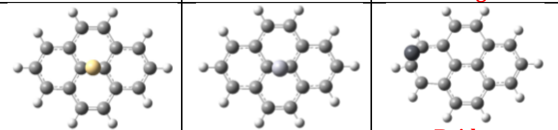 Bridge | Bridge | Bridge | AC-8 | 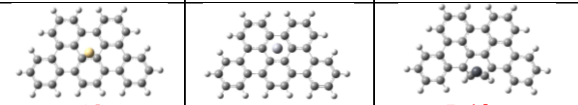 Bridge | Hollow | Bridge |
| ZZ-7 | 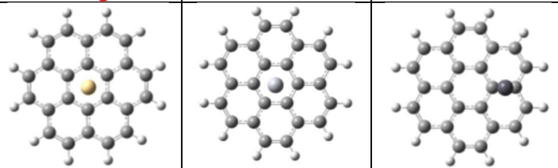 Hollow | Hollow | Bridge | AC-10 | 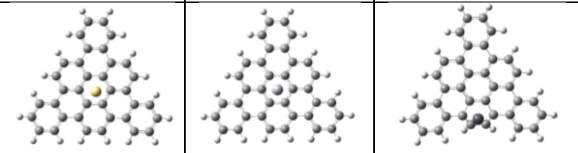 Hollow | Hollow | Bridge |
| ZZ-10 | 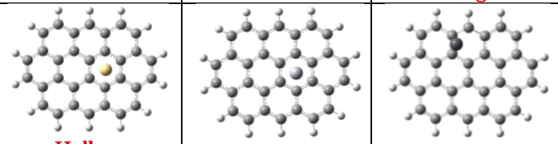 Hollow | Hollow | Bridge | AC-13 | 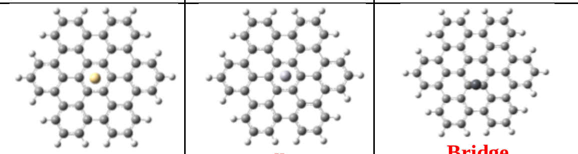 Hollow | Hollow | Bridge |
| ZZ-14 | 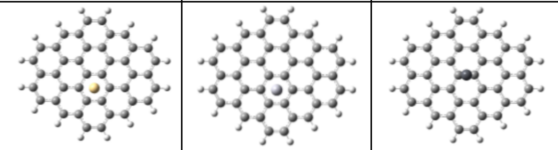 Hollow | Hollow | Bridge | AC-17 | 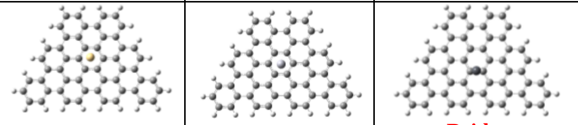 Hollow | Hollow | Bridge |
| ZZ-22 | 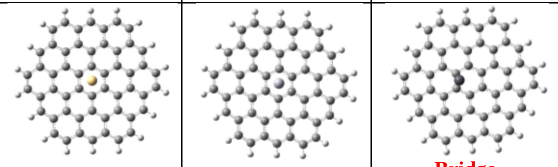 Hollow | Hollow | Bridge | AC-19 | 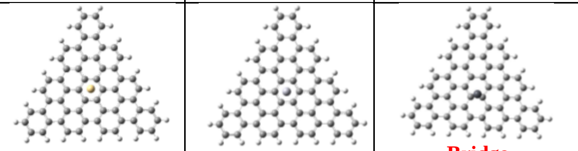 Hollow | Hollow | Bridge |

**Table 3. Structures of zigzag edged and armchair-edged GQDs interacting with divalent heavy metal ions.** "ZZ" and "AC" denotes a kind of edge, while the following integer corresponds to the number of hexagonal rings in GQD.

| ZZ-GQD | Structure | | | AC-GQD | Structure | | |
|---|---|---|---|---|---|---|---|
| | $Cd^{2+}$ | $Hg^{2+}$ | $Pb^{2+}$ | | $Cd^{2+}$ | $Hg^{2+}$ | $Pb^{2+}$ |
| ZZ-1 | 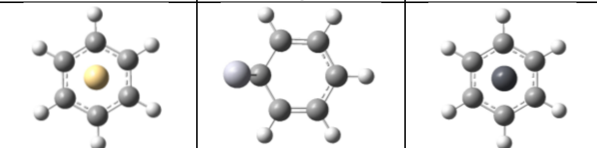 Hollow | Top | Hollow | AC-4 | 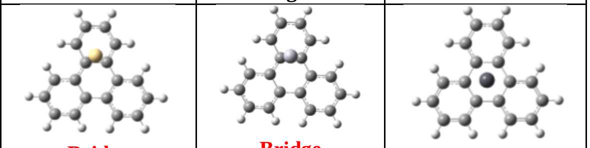 Bridge | Bridge | Hollow |
| | | | | AC-6 | 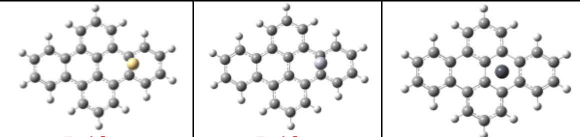 Bridge | Bridge | Hollow |
| ZZ-4 | 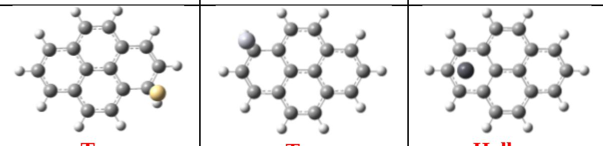 Top | Top | Hollow | AC-8 | 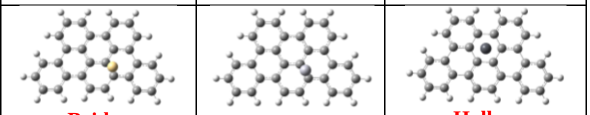 Bridge | Bridge | Hollow |
| ZZ-7 | 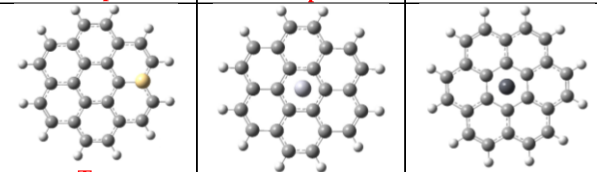 Top | Hollow | Hollow | AC-10 | 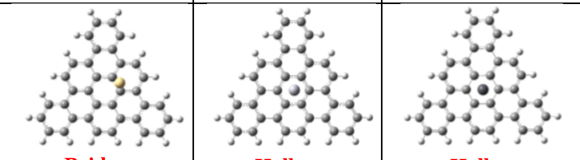 Bridge | Hollow | Hollow |
| ZZ-10 | 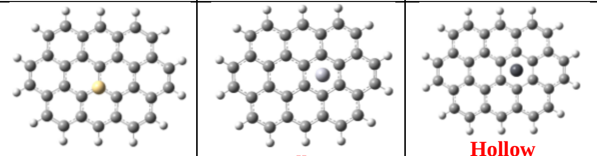 Top | Hollow | Hollow | AC-13 | 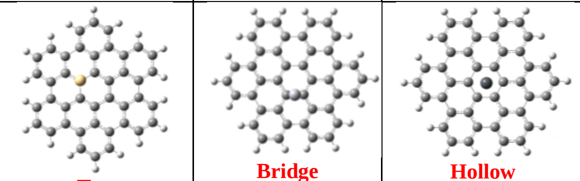 Top | Bridge | Hollow |
| ZZ-14 | 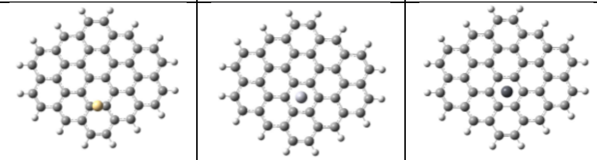 Bridge | Hollow | Hollow | AC-17 | 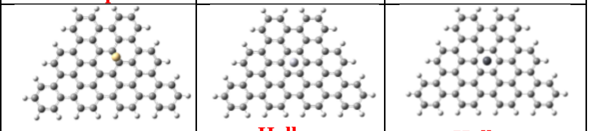 Bridge | Hollow | Hollow |
| ZZ-22 | 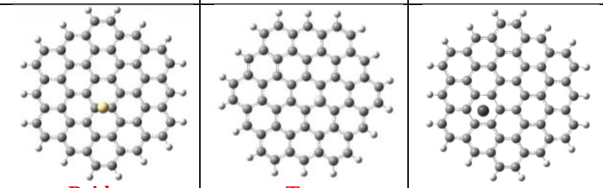 Bridge | Top | Hollow | AC-19 | 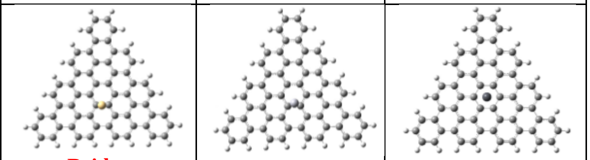 Bridge | Bridge | Hollow |

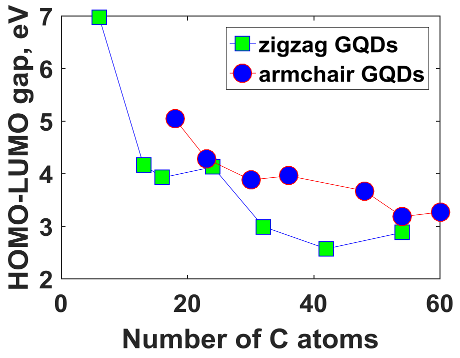


Figure S1 Dependence of the HOMO-LUMO gap of the zigzag-edged and armchair-edged graphene quantum dots on the size of the quantum dot

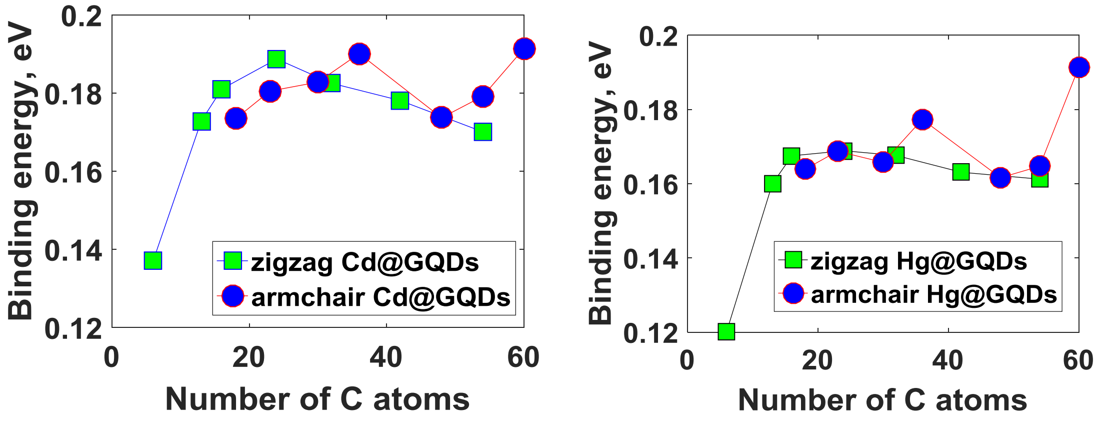


Figure S2 Dependence of the binding energy of (left panel) Cd and (right panel) Hg adsorbed on the surface of zigzag-edged and armchair-edged graphene quantum dots on the size of the quantum dot.

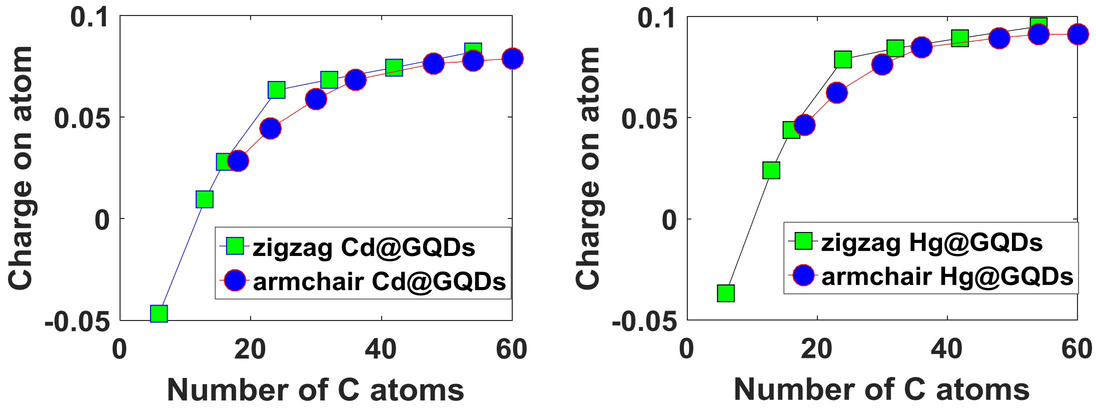


Figure S3 Dependence of the magnitude of Mulliken charge on (left panel) Cd and (right panel) Hg adsorbed on the surface of zigzag-edged and armchair-edged graphene quantum dots on the size of the quantum dot. The positive charges on atoms imply these adatoms are electron-donating species and charge transfer occurs from metal atom to GQD.

**Table S4 Parameters of the Zigzag-edged Graphene Quantum dots after interaction with neutral lead atoms.**

| Zigzag Graphene Quantum Dots | Binding energy, eV | Charge on HM | HOMO-LUMO gap, eV |
|---|---|---|---|
| Pb-Benzene $C_6H_6$ | 0.4670 | 0.11 | 0.99 |
| Pb-$C_{13}H_9$ | 1.9117 | 0.30 | 2.64 |
| Pb-pyrene $C_{16}H_{10}$ | 0.7065 | 0.36 | 1.68 |
| Pb-coronene $C_{24}H_{12}$ | 0.2103 | 0.11 | 0.75 |
| Pb-ovalene $C_{32}H_{14}$ | 0.6572 | 0.50 | 1.43 |
| Pb-Circumpyrene $C_{42}H_{16}$ | 0.2993 | 0.34 | 0.81 |
| Pb-Circumcoronene $C_{54}H_{18}$ | 0.1991 | 0.19 | 0.73 |

**Table S5 Parameters of the Armchair-edged Graphene Quantum dots after interaction with neutral lead atoms.**

| Armchair Graphene Quantum Dots | Binding energy, eV | Charge on HM | HOMO-LUMO gap, eV |
|---|---|---|---|
| Pb-triphenylene $C_{18}H_{12}$ | 0.3808 | 0.27 | 1.24 |
| Pb-$C_{23}H_{14}$ | 0.3557 | 0.27 | 1.17 |
| Pb-Tribenzo (b,n,pqr)perylene $C_{30}H_{16}$ | 0.4994 | 0.57 | 2.40 |
| Pb-Tribenzo(A,D,G)Coronene $C_{36}H_{18}$ | 0.4602 | 0.56 | 2.35 |
| Pb-HexabenzoCoronene $C_{48}H_{24}$ | 0.2878 | 0.27 | 0.90 |
| Pb-$C_{54}H_{22}$ | 0.2461 | 0.23 | 0.85 |
| Pb-$C_{60}H_{26}$ | 0.2894 | 0.29 | 0.88 |

**Table S6 Electronic transitions responsible for the adsorption peaks of the zigzag and armchair graphene quantum dots after complexation with divalent metal ions.**

| No. | Energy ($cm^{-1}$) | Wavelength (nm) | Osc. Strength | Symmetry | Major contribs | Minor contribs |
|---|---|---|---|---|---|---|
| **$Cd^{2+}$ on Armchair Graphene Quantum Dots ($C_{54}H_{22}$)** | | | | | | |
| 1 | 3245.57 | 3081.11 | 0.0005 | Singlet-A | H-1->LUMO (95%) | HOMO->LUMO (5%) |
| 2 | 4990.15 | 2003.94 | 0.0275 | Singlet-A | HOMO->LUMO (114%) | H-1->LUMO (5%) |
| 3 | 6629.07 | 1508.50 | 0.0498 | Singlet-A | H-2->LUMO (93%) | H-4->LUMO (7%) |
| 4 | 11624.8 | 860.22 | 0.0109 | Singlet-A | H-5->LUMO (85%), H-3->LUMO (13%) | |
| 5 | 11970.8 | 835.36 | 0.1191 | Singlet-A | H-4->LUMO (86%) | H-6->LUMO (3%), H-2->LUMO (7%), H-1->L+1 (3%) |
| 6 | 13253.3 | 754.52 | 0.5419 | Singlet-A | H-5->LUMO (12%), H-3->LUMO (85%) | |
| 7 | 13793.6 | 724.96 | 0.0024 | Singlet-A | H-6->LUMO (94%) | H-7->LUMO (2%), H-4->LUMO (3%) |
| 8 | 16185.9 | 617.82 | 0.0072 | Singlet-A | H-7->LUMO (97%) | |
| 9 | 17499.8 | 571.43 | 0.0026 | Singlet-A | H-8->LUMO (98%) | |
| 10 | 19058.8 | 524.68 | 0.0003 | Singlet-A | H-9->LUMO (97%) | |
| 11 | 19310.5 | 517.85 | 0.0006 | Singlet-A | H-10->LUMO (96%) | |
| 12 | 20321.9 | 492.07 | 0.0312 | Singlet-A | HOMO->L+1 (88%) | H-11->LUMO (9%) |
| **$Cd^{2+}$ on Zigzag Graphene Quantum Dots ($C_{54}H_{18}$)** | | | | | | |
| 1 | 3882.75 | 2575.49 | 0.0162 | Singlet-A | H-1->LUMO (121%) | |
| 2 | 9030.18 | 1107.39 | 0.0001 | Singlet-A | H-3->LUMO (99%) | |
| 3 | 9899.64 | 1010.13 | 0.0484 | Singlet-A | H-4->LUMO (94%) | H-5->LUMO (5%) |
| 4 | 12016.0 | 832.22 | 0.2318 | Singlet-A | H-2->LUMO (90%), HOMO->L+1 (11%) | |
| 5 | 15812.4 | 632.41 | 0.1198 | Singlet-A | H-5->LUMO (90%) | H-4->LUMO (4%), HOMO->L+2 (4%) |
| 6 | 17857.1 | 560.00 | 0.0002 | Singlet-A | H-6->LUMO (97%) | |
| 7 | 18573.3 | 538.40 | 0.0198 | Singlet-A | H-1->L+1 (96%) | HOMO->L+2 (2%) |

| | | | | | | |
|---|---|---|---|---|---|---|
| 8 | 20410.6 | 489.93 | 0.1804 | Singlet-A | H-1->L+2 (59%), HOMO->L+1 (37%) | H-2->LUMO (3%) |
| **$Hg^{2+}$ on Armchair Graphene Quantum Dots ($C_{54}H_{22}$)** | | | | | | |
| 1 | 3222.99 | 3102.70 | 0.0004 | Singlet-A | HOMO->LUMO (100%) | |
| 2 | 6521.79 | 1533.31 | 0.0004 | *Singlet-A* | H-2->LUMO (100%) | |
| 3 | 6783.92 | 1474.07 | 0.0532 | Singlet-A | H-4->LUMO (10%), H-1->LUMO (92%) | |
| 4 | 11713.5 | 853.70 | 0.0059 | Singlet-A | H-5->LUMO (91%) | H-3->LUMO (8%) |
| 5 | 11989.4 | 834.06 | 0.1348 | Singlet-A | H-4->LUMO (84%), H-1->LUMO (10%) | H-6->LUMO (3%), HOMO->L+1 (3%) |
| 6 | 13612.2 | 734.63 | 0.63 | Singlet-A | H-3->LUMO (90%) | H-5->LUMO (7%), HOMO->L+2 (2%) |
| 7 | 13873.5 | 720.79 | 0.0039 | Singlet-A | H-6->LUMO (93%) | H-7->LUMO (3%), H-4->LUMO (3%) |
| 8 | 16125.4 | 620.13 | 0.0095 | Singlet-A | H-7->LUMO (96%) | |
| 9 | 17398.9 | 574.74 | 0.0042 | Singlet-A | H-8->LUMO (98%) | |
| 10 | 18982.2 | 526.80 | 0.0001 | Singlet-A | H-9->LUMO (97%) | |
| 11 | 19339.5 | 517.07 | 0.0019 | Singlet-A | H-10->LUMO (96%) | |
| 12 | 20892.1 | 478.64 | 0.021 | Singlet-A | H-11->LUMO (90%) | H-1->L+1 (5%) |
| **$Hg^{2+}$ on Zigzag Graphene Quantum Dots ($C_{54}H_{18}$)** | | | | | | |
| 1 | 6950.07 | 1438.83 | 0.0019 | Singlet-A | H-1->LUMO (100%) | |
| 2 | 8948.72 | 1117.47 | 0.0001 | Singlet-A | H-3->LUMO (99%) | |
| 3 | 9900.45 | 1010.05 | 0.0536 | Singlet-A | H-4->LUMO (93%) | H-5->LUMO (6%) |
| 4 | 12193.4 | 820.10 | 0.2465 | Singlet-A | H-2->LUMO (88%), HOMO->L+1 (12%) | |
| 5 | 15980.2 | 625.77 | 0.1457 | Singlet-A | H-5->LUMO (89%) | H-4->LUMO (5%), HOMO->L+2 (5%) |
| 6 | 21457.5 | 466.03 | 0.824 | Singlet-A | H-2->LUMO (12%), HOMO->L+1 (85%) | |
| **$Pb^{2+}$ on Armchair Graphene Quantum Dots ($C_{54}H_{22}$)** | | | | | | |
| 1 | 10046.4 | 995.37 | 0.0021 | Singlet-A | HOMO->LUMO (96%) | H-2->LUMO (3%) |
| 2 | 10791.6 | 926.63 | 0.0017 | Singlet-A | H-1->LUMO (25%), HOMO->L+1 (74%) | |
| 3 | 11774.0 | 849.32 | 0.0002 | Singlet-A | H-1->L+1 (98%) | |
| 4 | 12553.2 | 796.60 | 0.0203 | Singlet-A | H-2->LUMO (96%) | HOMO->LUMO (3%) |
| 5 | 12592.7 | 794.10 | 0.0137 | Singlet-A | H-2->L+1 (88%) | H-1->LUMO (6%), HOMO->L+1 (5%) |
| 6 | 13551.7 | 737.91 | 0.024 | Singlet-A | H-2->L+1 (11%), H-1->LUMO (68%), HOMO->L+1 (20%) | |
| 7 | 16750.5 | 596.99 | 0.001 | Singlet-A | H-3->LUMO (99%) | |
| 8 | 16844.0 | 593.68 | 0.0191 | Singlet-A | H-3->L+1 (97%) | |
| 9 | 17451.4 | 573.01 | 0.0015 | Singlet-A | HOMO->L+2 (95%) | |
| 10 | 18876.5 | 529.75 | 0.066 | Singlet-A | H-4->LUMO (10%), H-1->L+4 (12%), HOMO->L+3 (73%) | |
| 11 | 19058.8 | 524.68 | 0.0004 | Singlet-A | H-1->L+2 (97%) | |
| 12 | 19219.3 | 520.30 | 0.0184 | Singlet-A | H-4->LUMO (83%), HOMO->L+3 (12%) | |
| **$Pb^{2+}$ on Zigzag Graphene Quantum Dots ($C_{54}H_{18}$)** | | | | | | |
| 1 | 6817.80 | 1466.74 | 0.0006 | Singlet-A | H-1->LUMO (88%), HOMO->L+1 (11%) | |
| 2 | 6908.13 | 1447.56 | 0.0047 | Singlet-A | HOMO->LUMO (98%) | |
| 3 | 7225.92 | 1383.90 | 0.0085 | Singlet-A | H-1->LUMO (11%), HOMO->L+1 (88%) | |
| 4 | 8415.58 | 1188.27 | 0.0314 | Singlet-A | H-1->L+1 (96%) | |
| 5 | 14742.2 | 678.32 | 0.0027 | Singlet-A | H-2->LUMO (91%) | HOMO->L+2 (7%) |
| 6 | 14940.6 | 669.31 | 0.0003 | Singlet-A | HOMO->L+2 (93%) | H-2->LUMO (7%) |
| 7 | 15244.6 | 655.96 | 0.0028 | Singlet-A | H-2->L+1 (92%) | H-3->LUMO (7%) |
| 8 | 16089.1 | 621.53 | 0.0006 | Singlet-A | H-4->LUMO (91%) | H-3->L+1 (6%) |
| 9 | 16237.5 | 615.85 | 0.0022 | Singlet-A | H-3->L+1 (91%) | H-4->LUMO (6%) |
| 10 | 16239.1 | 615.79 | 0.0015 | Singlet-A | H-4->L+1 (85%), H-3->LUMO (12%) | |
| 11 | 16786.8 | 595.70 | 0.0088 | Singlet-A | H-3->LUMO (69%), HOMO->L+3 (12%) | H-4->L+1 (8%), H-2->L+1 (6%), H-1->L+4 (4%) |

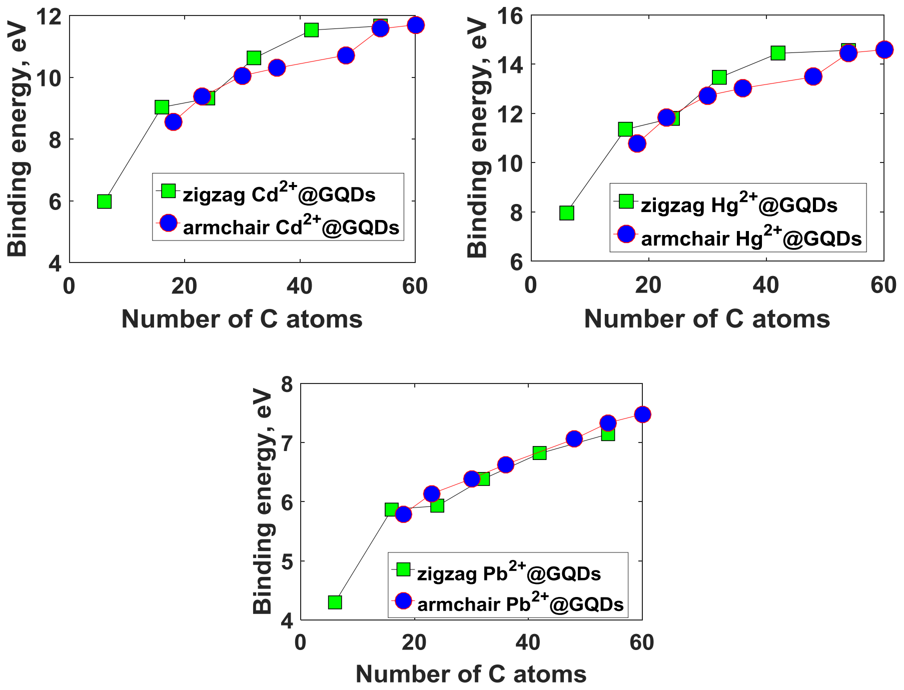


Figure S4 Dependence of the binding energy of (top left panel) $Cd^{2+}$, (top right panel) $Hg^{2+}$ and (bottom panel) $Pb^{2+}$ adsorbed on the surface of zigzag-edged and armchair-edged graphene quantum dots on the size of the quantum dot.

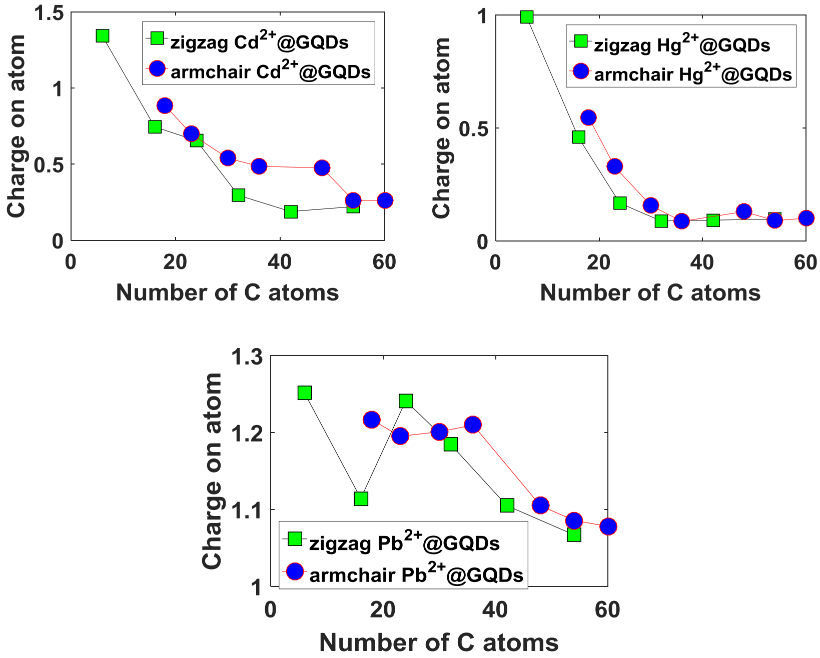


Figure S5 Dependence of the magnitude of Mulliken charge on (top left panel) $Cd^{2+}$, (top right panel) $Hg^{2+}$ and (bottom panel) $Pb^{2+}$ adsorbed on the surface of zigzag-edged and armchair-edged graphene quantum dots on the size of the quantum dot. The deviation of the magnitude of the charge from +2 towards lower values of positive charge imply the HM ions are hole-donating species and charge transfer occurs from GQD atom to metal.

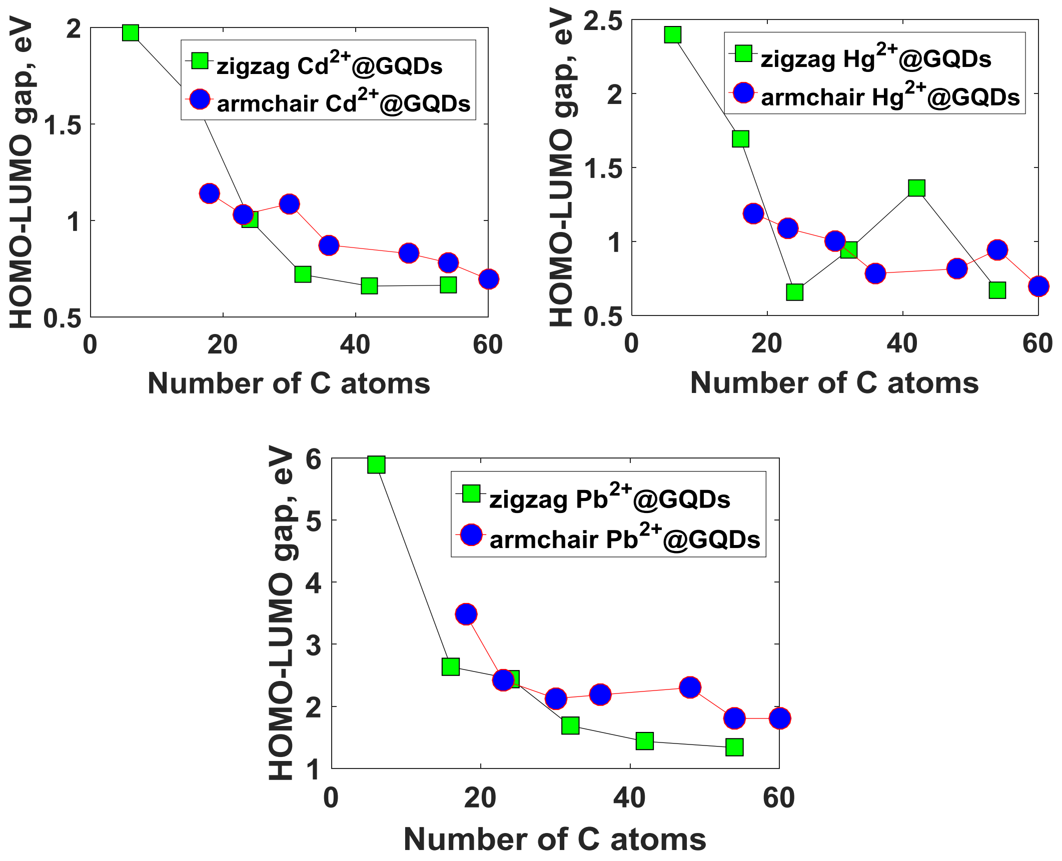


Figure S6 Dependence of the binding energy of (top left panel) $Cd^{2+}$, (top right panel) $Hg^{2+}$ and (bottom panel) $Pb^{2+}$ adsorbed on the surface of zigzag-edged and armchair-edged graphene quantum dots on the size of the quantum dot.
